# The *integripennis* species group of *Geocharidius* Jeannel, 1963 (Carabidae, Bembidiini, Anillina) from Nuclear Central America: a taxonomic review with notes about biogeography and speciation

**DOI:** 10.3897/zookeys.443.7880

**Published:** 2014-09-30

**Authors:** Igor M. Sokolov, David H. Kavanaugh

**Affiliations:** 1 Department of Entomology, California Academy of Sciences, Golden Gate Park, 55 Music Concourse Drive, San Francisco, CA 94118, USA

**Keywords:** Coleoptera, Adephaga, Carabidae, Bembidiini, Anillina, *Geocharidius*, new species, Nuclear Central America, Motagua Fault Zone, biogeography, sympatric speciation

## Abstract

Our review recognizes 15 species of the *integripennis* species group of *Geocharidius* from Nuclear Central America, include three species previously described (*Geocharidius gimlii* Erwin, *Geocharidius integripennis* (Bates) and *Geocharidius zullinii* Vigna Taglianti) and 12 described here as new. They are: *Geocharidius andersoni* **sp. n.** (type locality: Chiapas, Chiapas Highlands, Cerro Huitepec) and *Geocharidius vignatagliantii* **sp. n.** (type locality: Chiapas, Motozintla, Sierra Madre de Chiapas, Benito Juárez) from Mexico; *Geocharidius antigua* **sp. n.** (type locality: Sacatepéquez, 5 km SE of Antigua), *Geocharidius balini* **sp. n.** (type locality: Suchitepéquez, 4 km S of Volcan Atitlán), *Geocharidius erwini* **sp. n.** (type locality: Quiché Department, 7 km NE of Los Encuentros), *Geocharidius jalapensis* **sp. n.** (type locality: Jalapa Department, 4 km E of Mataquescuintla), *Geocharidius longinoi*, **sp. n.** (type locality: El Progreso Department, Cerro Pinalón), and *Geocharidius minimus* **sp. n.** (type locality: Sacatepéquez Department, 5 km SE of Antigua) from Guatemala; and *Geocharidius celaquensis* **sp. n.** (type locality: Lempira Department, Celaque National Park), *Geocharidius comayaguanus* **sp. n.** (type locality: Comayagua Department, 18 km ENE of Comayagua), *Geocharidius disjunctus* **sp. n.** (type locality: Francisco Morazán, La Tigra National Park), and *Geocharidius lencanus* **sp. n.** (type locality: Lempira Department, Celaque National Park) from Honduras. For all members of the group, adult structural characters, including male and female genitalia, are described, and a taxonomic key for all members of the *integripennis* species group is presented based on these characters. Behavioral and biogeographical aspects of speciation in the group are discussed, based on the morphological analysis. In all cases of sympatry, pairs of closely related species show greater differences in sizes than pairs of more remotely related species. *Integripennis* group species occupy six different montane areas at elevations above 1300m, with no species shared among them. Major faunal barriers in the region limiting present species distributions include the Motagua Fault Zone and a gap between the Guatemalan Cordillera volcanic chain and the Honduran Interior Highlands no higher than 900m in elevation. Highest species diversity is in the Guatematan Cordillera (six species), second highest in the Honduran Interior Highlands area (four species).

## Introduction

*Geocharidius* Jeannel, 1963 was established for a Guatemalan species, *Geocharidius integripennis* (Bates), described in “Biologia Centralia-Americana” (Bates 1882). Jeannel’s description of *Geocharidius* omitted or misinterpreted several important morphological details, leading Vigna Taglianti (1973) to re-describe the genus on the basis of the two species, *Geocharidius integripennis* and *Geocharidius zullinii* Vigna Taglianti, known to him at that time. Providing new evidence, Vigna Taglianti (l.c.) proposed a new phyletic lineage for *Geocharidius*, which had been placed by Jeannel (1963) in a lineage with the Mediterranean *Geocharis* Ehlers and *Rhegmatobius* Jeannel. The new lineage integrated the New World anillines of Jeannel’s “scotodipnienne” stock of genera (i.e. those taxa, members of which have a mental tooth along with the umbilicate series of elytral pores of type B (Jeannel 1937), where pores 7 and 8 and 8 and 9 are separated from each other by equal distances. According to Vigna Taglianti (1973) this lineage included also *Mexanillus* Vigna Taglianti, described in the same paper, and perhaps also *Mystroceridius* Reichardt (1970) from the Galapagos Islands. Since then, several new genera of the “scotodipnienne” stock of anilline genera have been described from the New World (Zaballos 1997; Mateu and Etonti 2002). At present, *Geocharidius* includes 5 species (Lorenz 2005; Zaballos 2004), four of which are limited in their distribution to Guatemala (Erwin 1982). Ecologically, representatives of *Geocharidius* are adapted for life in forest litter, and these beetles are comparatively easy to collect using litter sifting techniques.

From 2008 to 2011, the “Leaf Litter Arthropods of Mesoamerica” (LLAMA) project (http://llama.evergreen.edu/) generated the first significant samples of the leaf litter invertebrate fauna of Mesoamerica (including southern Mexico). This project focused on sampling ants and weevils from the litter layer of the tropical forest floor, but it also sampled many different non-target taxa, including many litter anillines. By 2012, the second author (DHK) had assembled on loan most available material representing Mesoamerican Anillina at the California Academy of Sciences, San Francisco. Several hundred specimens of the subtribe were borrowed from the collections of the six institutions noted below. This material served as the basis of and inspiration for the current report. In this paper, we present the results of a taxonomic study of one intrageneric group of species of *Geocharidius*, the *integripennis* species group.

## Materials and methods

This study is based on examination of 455 specimens belonging to the *integripennis* group of species of *Geocharidius*, which includes 15 species. Material was borrowed from and/or is deposited in the following institutions, identified in the text by the following associated codens:

**CAS** California Academy of Sciences, 55 Music Concourse Drive, San Francisco, California 94118 (D. H. Kavanaugh, Curator)

**CMNC** Canadian Museum of Nature, Entomology, P.O. Box 3443, Station D, Ottawa, Ontario, Canada, K1P 6P4 (R. S. Anderson, Curator)

**CMNH** Carnegie Museum of Natural History, Pittsburgh, Pennsylvania, U.S.A. 15213 (R. L. Davidson, Collections Manager)

**KUNHM** University of Kansas Natural History Museum, 1345 Jayhawk Blvd., Lawrence, Kansas, 66045-7593USA (Z. Falin, Collection Manager)

**MNHN** Muséum national d’Histoire naturelle de Paris, 57 Rue Cuvier, Paris, 75005, France (T. Deuve and A. Taghavian)

**NMNH** Department of Entomology, United States National Museum of Natural History, Smithsonian Institution, Washington, D. C., U.S.A. 20013-7012 (T. L. Erwin, Curator)

Verbatim label data are given for type specimens of all newly described taxa, with label breaks indicated by a slash (“\”). For a series of KUNHM specimens with the same geographical labels but differing in various barcode numbers only, these numbers were replaced in the text by periods of ellipsis (“…”).

Measurements. All specimens were measured electronically using a Leica M420 macroscope equipped with a Syncroscopy AutoMontage photomicroscopy system (SYNCROSCOPY, Synoptics Ltd.). Measurements for various body parts are encoded as follows: LH = length of head, measured along midline from anterior margin of labrum to a virtual line connecting posterior supraorbital setae; WH = width of head, at level of anterior supraorbital setae; WPm = maximum width across pronotum; WPa = width across anterior angles of pronotum; WPp = width across posterior angles of pronotum; LP = length of pronotum from base to apex along midline; WE = width of elytra, at level of 4^th^ umbilicate setae; LE = length of the elytra, from apex of scutellum to apex of left elytron; SBL = standardized body length, a sum of LH, LP and LE. Measurements of SBL are given in millimeters; others are presented as eight ratios: mean widths-WH/WPm and WPm/WE and body parts-WPa/WPp, WPm/WPp, WPm/LP, WE/LE, LE/SBL and WE/SBL. All values are given as mean ± standard deviation.

Illustrations. Digital photographs of the dorsal habitus of new species were taken with the AutoMontage system using a Leica M420 macroscope. Line drawings of selected body parts were made using grids on a Labomed Lx400 compound microscope. Scanning electron micrographs were made with coating on a LEO 1450VP SEM. Diagrams were prepared using Statistica 6.0. (StatSoft Inc.).

Dissections. Dissections were made using standard techniques. Genitalia were dissected from abdomens of specimens previously softened in boiling water for 20–30 minutes. Contents of the abdomen were cleared using boiling 10% KOH for 2-3 minutes to remove internal tissues, and then washed in hot water before examination. After examination, genitalia were mounted on plastic transparent boards in dimethylhydantoin formaldehyde resin (DMHF) and pinned beneath the specimen. In some species, investigation of body parts was undertaken in the following way. The whole specimen was cleared using boiling 10% KOH for ~5 minutes, then washed and dissected. Disassembled body parts from a single specimen were placed on plastic transparent cardboard, properly oriented, mounted in DMHF and pinned together with the specimen labels.

Type material. The authors had no opportunity to investigate type material of the Mexican species of Anillina described by A. Vigna Taglianti. The identification of *Geocharidius zullinii* was made only on the basis of the original description of the species (Vigna Taglianti 1973). Types of the Guatemalan species of *Geocharidius* described by T. L. Erwin in his revision of Central American Bembidiini (Erwin 1982) were examined. All paratypes listed in the treatments for new species have been labeled with appropriate yellow paratypes labels, which have not been included in the verbatim label data provided for each specimen.

Terms. Terms used in the paper are largely of general use and follow the literature (Ball and Bousquet 2000; Ball and Shpeley 2005, 2009; Erwin 1974; Jeannel 1963), except those for ventral surface structures, terms for which follow the Handbook of Zoology (Lawrence et al. 2010). We use the term “dorsal sclerites” (eg. Fig. 9A, D, E and H) to refer to a complex of more or less sclerotized figs and/or flagellum-like pieces in the dorsobasal region of the retracted internal sac of the male median lobe. These sclerotized elements are highly varied in their size, shape and number and/or degree of fusion among males of different species of this species group, and we have not yet distinguished individual homologies among the varied elements. However, we distinguish this complex of sclerites as a group from the “ventral sclerites” complex found in males of many species of *Anillinus* Casey (1918) along with the dorsal sclerite complex (eg., see Sokolov 2014, fig. 6K, vsc). We defined Nuclear Central America as the region between the Isthmus of Tehuantepec and the Nicaraguan Depression (Schuchert, 1935).

Species ranking. Species recognition is in accordance with our previous approach (Sokolov et al. 2004), except for cases explained in the text.

Arrangement of taxa in the text. Taxonomic treatments of species are arranged separately by country for the region (i.e. Mexico, Guatemala and Honduras) consistent with the geographical distinctions made in our key to species. For each country, species treatments are arranged in alphabetical order..

Descriptions. The scheme of description generally follows that of Ball and Shpeley (2005, 2009).

Map. The map (Fig. 22) was downloaded from the web-site: http://www.maps-for-free.com/ and adjusted using Adobe Photoshop® software.

## Taxonomy

### Geocharidius

Taxon classificationAnimaliaColeopteraCarabidae

Jeannel

Geocharidius Jeannel, 1963: 107 (type species *Anillus integripennis* Bates, 1882, by original designation)

#### Recognition.

The members of this genus are distinguished from those of the other North and Central American Anillina by the following combination of characters: frontal area of head with small median tubercle; maxillary palps with palpomere 4 shorter than 1/4 that of palpomere 3; labial ligula without paraglossae, mentum and submentum separated by mental-submental suture; pronotum convex, with short vestiture throughout, including the areas forward of the lateral setae; elytra without fixed discal setae and with the 7^th^ and the 8^th^ and the 8^th^ and the 9^th^ pores of the umbilicate series separated by equal distances; metendoventrite linear without lateral arms; and intercoxal process between the hind legs widely triangular (Sokolov 2013).

#### Included taxa.

The species of *Geocharidius*, as treated at present (Lorenz 2005), are arranged in two groups, based on body form: those with a subdepressed form and those with a globose habitus (Erwin 1982). Species with members subdepressed in habitus (Fig. 2A–C) correspond to the type species of the genus and are treated below as the *integripennis* species group. Members of the genus with a globose habitus (Fig. 2D), like *Geocharidius phineus* Erwin, *Geocharidius romeoi* Erwin and similar undescribed species, are not treated in this report.

### The *integripennis* species group

**Figures 1.**
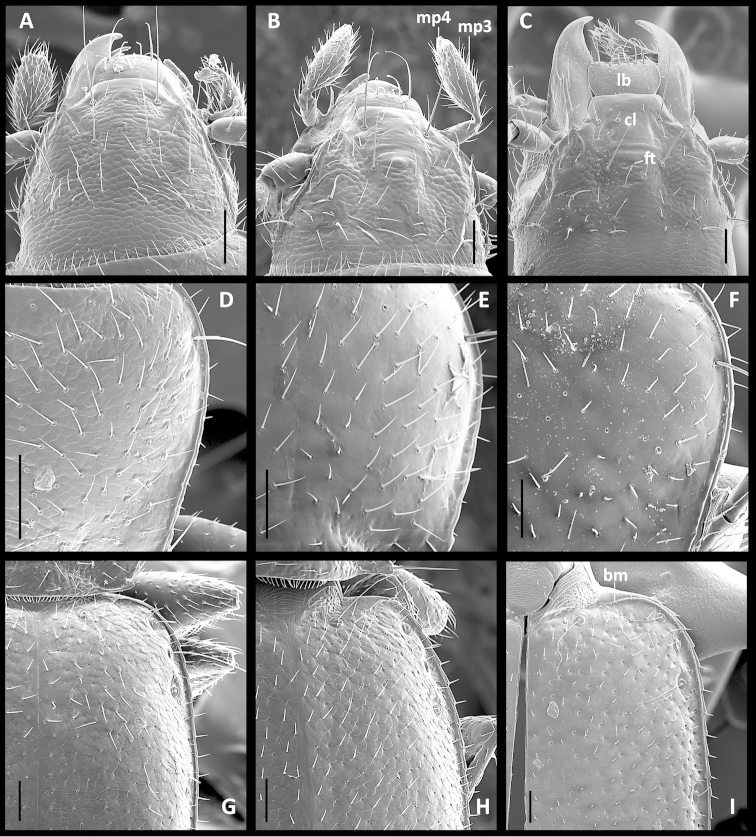
SEM illustrations of structural features of body parts of *Geocharidius* species. **A–C** head, dorsal aspect: **A** *Geocharidius balini* **B** *Geocharidius zullinii* **C** *G. andersoni.* **D–F** right half of pronotum, dorsal aspect: **D** *Geocharidius balini* **E** *Geocharidius zullinii* **F** *Geocharidius andersoni*. **G–I** basal part of right elytron: **G** *Geocharidius balini* **H** *Geocharidius zullinii* **I** *Geocharidius andersoni* Legend: bm – basal margin; cl – clypeus; ft – frontal tubercle; lb – labrum; mp3 – maxillary palpomere 3; mp4 – maxillary palpomere 4. Scale = 0.05mm.

**Figures 2.**
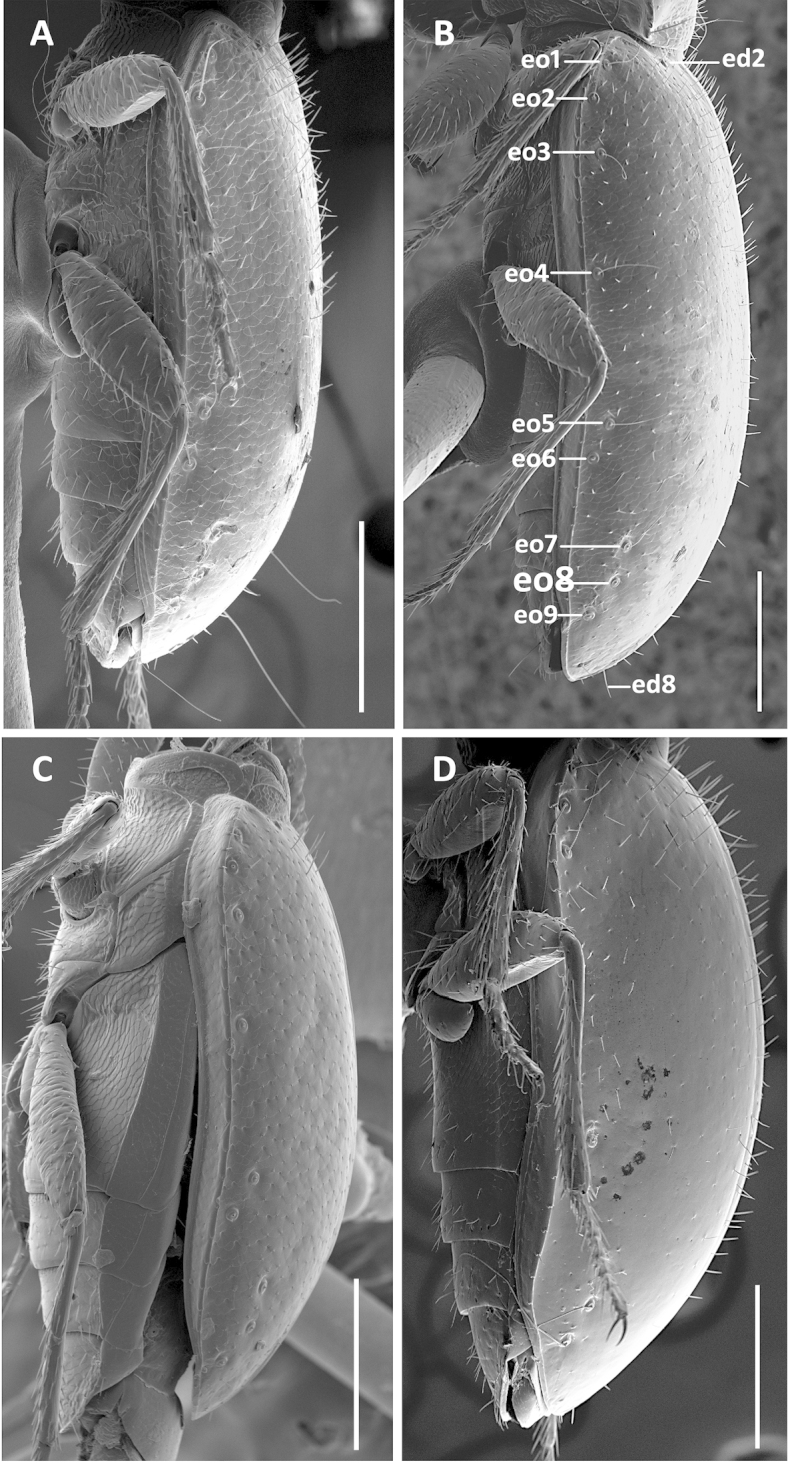
SEM illustrations of structural features and shape of elytra of *Geocharidius* species, left lateral aspect. **A** *Geocharidius erwini* **B** *Geocharidius jalapensis* **C** *Geocharidius comayaguanus* **D** *Geocharidius phineus*. Legend: ed2 – scutellar seta; ed8 – apical seta; eo1-9 – setae 1-9 from the umbilicate series. Scale = 0.2mm.

**Recognition.** Members of this group are distinguished from the other representatives of the genus by the following combination of external characters: head totally covered with microlines, microsculpture mesh pattern isodiametric (Figs 1A–C) and elytra only moderately convex (subdepressed) (Fig. 2A–C). Most species also have members with the elytra totally covered with microlines in form of isodiametric mesh pattern (Figs 1G–H), males with long copulatory sclerites of the internal sac (Figs 9, 13, 19) and females with simple, not bilobate, spermatheca (Figs 11, 17, 21).

**Description.** *Size.* SBL range 1.15-1.61 mm.

Habitus. Body form slightly to moderately convex, subparallel or slightly ovoid.

Color. Body monocolorous, brunneorufous or rufotestaceous, appendages testaceous.

**Figures 3.**
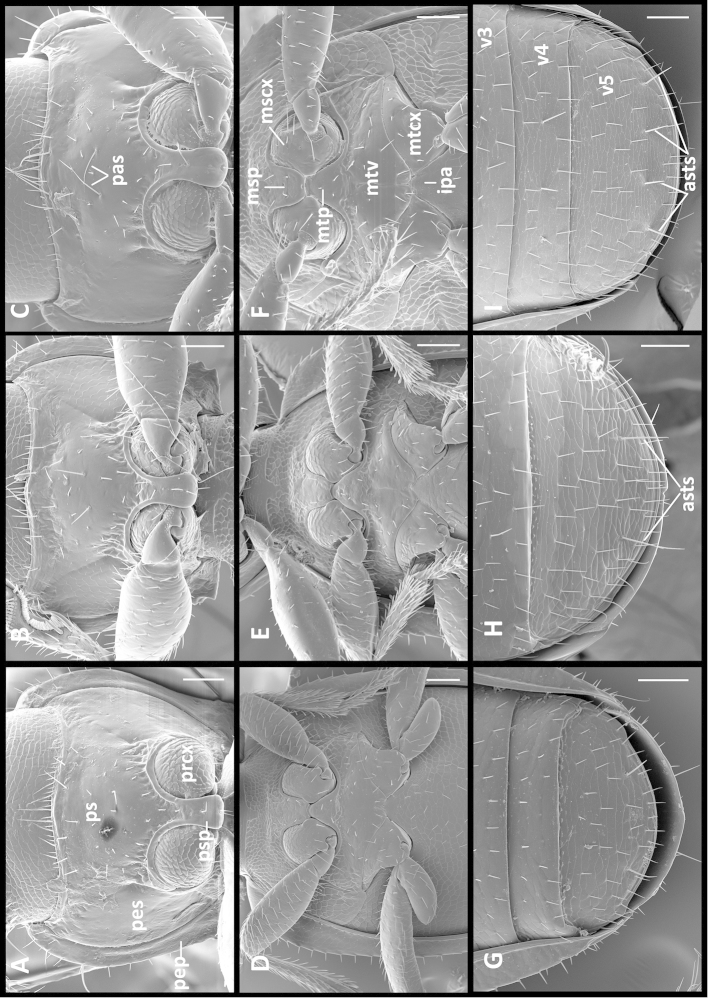
SEM illustrations of structural features of body parts of *Geocharidius* species. **A–C** prothorax, ventral aspect: **A** *Geocharidius zullinii* **B** *Geocharidius erwini* **C** *Geocharidius comayaguanus*. **D–F** pterothorax, ventral aspect: **D** *Geocharidius jalapensis* **E** *Geocharidius minimus* **F** *Geocharidius comayaguanus*. **G–I** abdominal ventrites 3-5: **G** *Geocharidius minimus*, male **H** *Geocharidius jalapensis*, male **I** *Geocharidius jalapensis* female. Legend: asts – abdominal sternal terminal seta; ipa –intercoxal process of abdominal ventrite 2; mscx – mesocoxa; msp – mesosternal process; mtcx – metacoxa; mtp – metasternal process; mtv – metaventrite; pas – prosternal ambulatory seta; pep – proepipleuron; pes – proepisternum; prcx – procoxa; ps – prosternum; psp – prosternal process; v3-v5 – abdominal ventrites 3-5. Scale = 0.05mm.

Microsculpture. Dorsal surface with polygonal sculpticells present on head in all species, also on elytra except in *Geocharidius andersoni* members with smooth elytra (Fig. 1I). Development of microsculpture on pronotum and proepisternum (pes) varied among different species (Figs 1D–F, Figs 3A–C).

Luster. Body surface shiny.

Macrosculpture. Body surface sparsely and finely punctate.

Vestiture. Body surface covered with sparse yellow setae of moderate and more or less equal length throughout.

Fixed setae. Primary head setae include one pair of clypeal, one pair of frontal and two pairs of supraorbital setae. Mentum (Fig. 5) with two pairs of long primary (paramedial [pms] and lateral [lms]) setae. Submentum (Fig. 5) with three groups of setae: two (Fig. 5D) to three (Fig. 5C) in medial row (mss), two (Fig. 5F) to three (Fig. 5C) in lateral rows (lss) and one pair of primary basal setae (prss). Elytra without discal setae (Fig. 2), but with scutellar (ed2) and apical setae (ed8) present. Last three (7^th^, 8^th^ and 9^th^) pores of umbilicate series (eo7, eo8 and eo9) in line and equally spread apart (Fig. 2B). Fifth abdominal ventrite (Fig. 3G–I) of male with one pair (Fig. 3H) and of female with two pairs (Fig. 3I) of abdominal setae along the posterior margin.

Head (Fig. 1A–C). Anterior margin of clypeus straight. Frontal area with small tubercle medially near frontoclypeal suture. Fronto-lateral carinae distinct and long.

Eyes. Absent.

Antennae. Submoniliform, with 11antennomeres, extended to about posterior margin of pronotum. Antennomeres 1 and 2 elongate, of approximately equal length and 1.4-1.5 times longer than antennomere 3, which is only slightly elongate and 1.1-1.2 times longer than antennomere 4. Antennomere 4 the shortest and 1.1.-1.2 times shorter than antennomere 5. Antennomeres 5 to 10 globose, antennomere 11 conical and 1.6-1.7 times longer than antennomere 10.

Labrum (Fig. 1A–C). Transverse with straight, entire anterior margin, with six setae apically, increasing in size from central pair outward.

**Figures 4.**
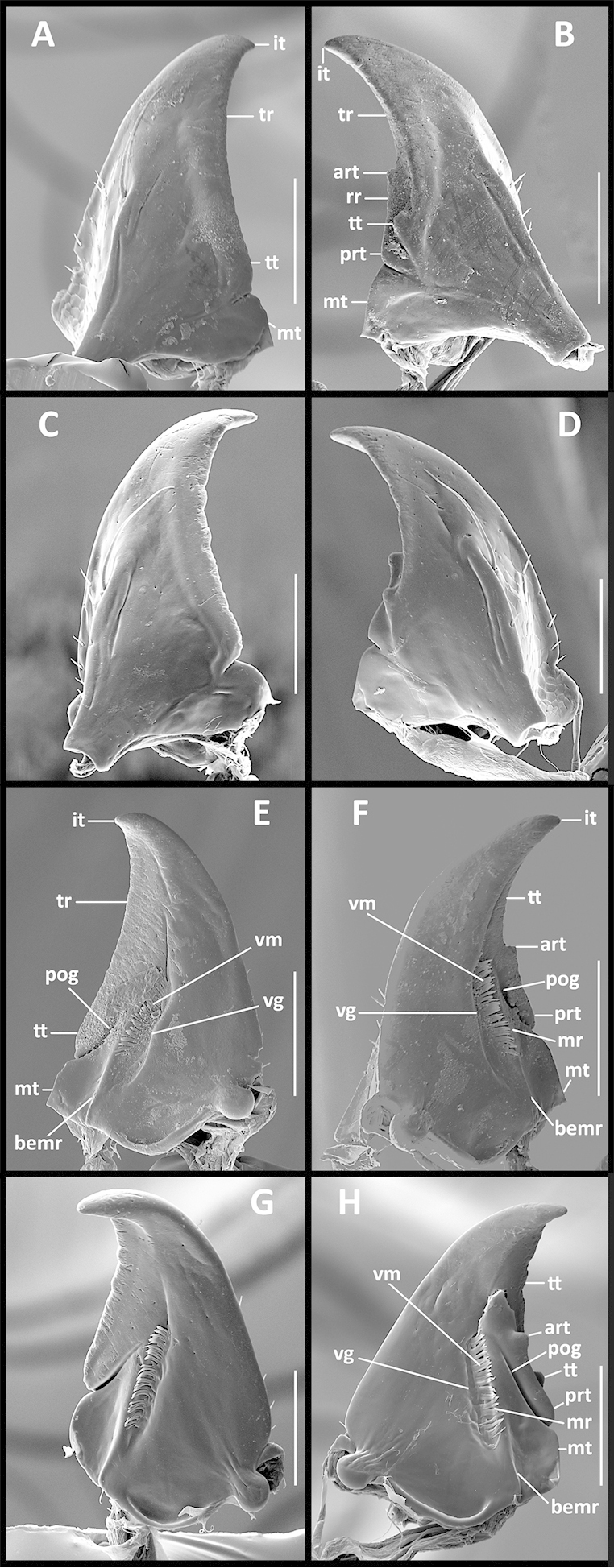
SEM illustrations of structural features of mandibles of *Geocharidius* species. **A–B** *Geocharidius zullinii*: dorsal aspect of left and right mandibles, respectively **C–D** *Geocharidius jalapensis*: dorsal aspect of left and right mandibles, respectively **E–F** *Geocharidius zullinii*:–ventral aspect of left and right mandibles, respectively **G–H** *Geocharidius jalapensis*: ventral aspect of left and right mandibles, respectively (right mandible with Λ-shaped crack apically from retinacular ridge). Legend: art – anterior retinacular tooth; bemr – molar ridge, basal extension; it – incisor tooth; mr – molar ridge; mt – molar tooth; pog – posterior occlusal grove; prt – posterior retinacular tooth; rr – retinacular ridge; tr – terebral ridge; tt – terebral tooth; vg – ventral groove; vm – microtrichia. Scale = 0.05mm.

Mandibles (Fig. 4). Right mandible with distinct anterior (art) and posterior (prt) retinacular, terebral (tt), and molar (mt) teeth. Left mandible with terebral and molar teeth only. Premolar teeth absent from both mandibles.

Maxillae. Cardo trianguloid, stipes with dorsal and ventral lobes, galea dimerous, lacinia standard for bembidiines. Palpus (Fig. 1A–B) with short 4^th^ palpomere, 0.2–0.25 length of palpomere 3.

**Figures 5.**
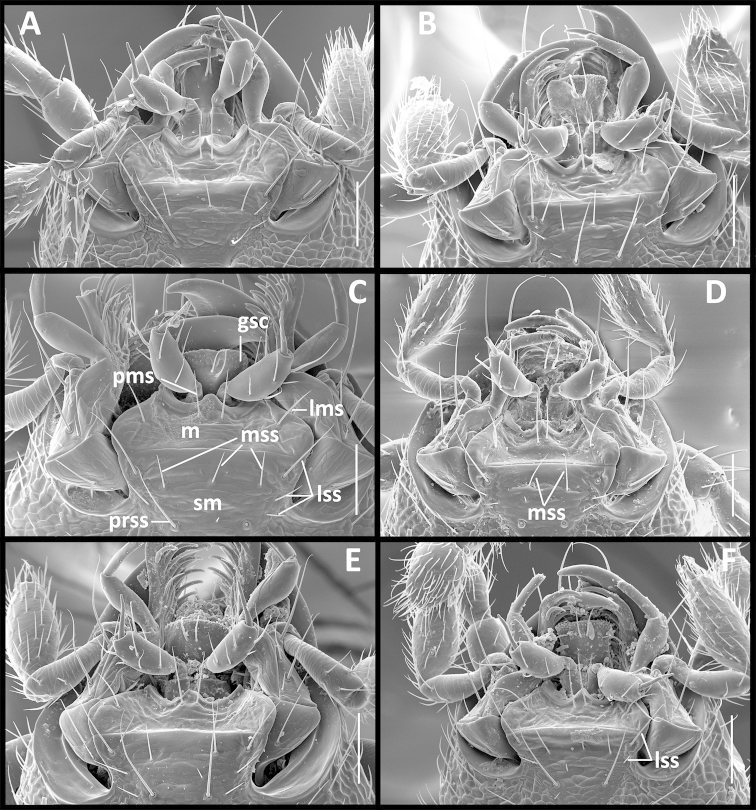
SEM illustrations of structural features of labial complex of *Geocharidius* species. **A** *Geocharidius erwini* **B** *Geocharidius balini* **C** *Geocharidius lencanus* **D** *Geocharidius zullinii* **E** *Geocharidius longinoi* **F** *Geocharidius minimus*. Legend: gsc – glossal sclerite; lms – lateral mental seta; lss – lateral submental seta; m – mentum; mss – medial submental seta; pms – paramedial mental seta; prss – primary basal submental seta; sm – submentum. Scale = 0.05mm.

Labium (Fig. 5). Mental tooth present; mentum (m) and submentum (sm) divided by mental-submental suture. Glossal sclerite (gsc) bisetose, without distinct paraglossae.

Prothorax. Pronotum cordiform, of moderate length (LP/SBL varied from 0.23 to 0.24 among species, LP/LE varied from 0.38 to 0.42 among species), moderately convex, not sinuate posteriorly. Basal margin of pronotum with slightly protruding medial portion. Anterior angles indistinct, broadly rounded. Posterior angles denticulate, with two or three small denticles anterior to angles. Prosternum (Fig. 3A–C, ps) slightly protruding at the anterior margin medially, there with a group of longer setae relative to other prosternal vestiture, also with a pair of long ambulatory sensor setae (pas) at the middle of sclerite. Prosternal intercoxal process unmargined, slightly dilated apically and obtusely truncate at apex, with a row of sparse setulae along midline.

Scutellum. Externally visible, triangular, with rounded apex.

Elytra. Moderate in length (LE/SBL varied from 0.57 to 0.60 among species), without visible interneurs. Basal margination varied (long in some species, extended halfway between humeral angle and scutellar pore (Fig. 1H–I, bm), very short in others, length about equal to diameter of basal setiferous pore socket (Fig. 1G)) but distinct in all species. Lateral elytral margin without subapical sinuation in apical half.

Hind wings. Absent.

Pterothoracic venter (Fig. 3D–F). Metaventrite (mtv) short, distance between meso-and metacoxae (mtcx) slightly less than the diameter of mesocoxa (mscx). Metanepisternum short, subquadrate, with anterior and outer margins of equal length. Metendoventrite linear without lateral arms.

**Figures 6.**
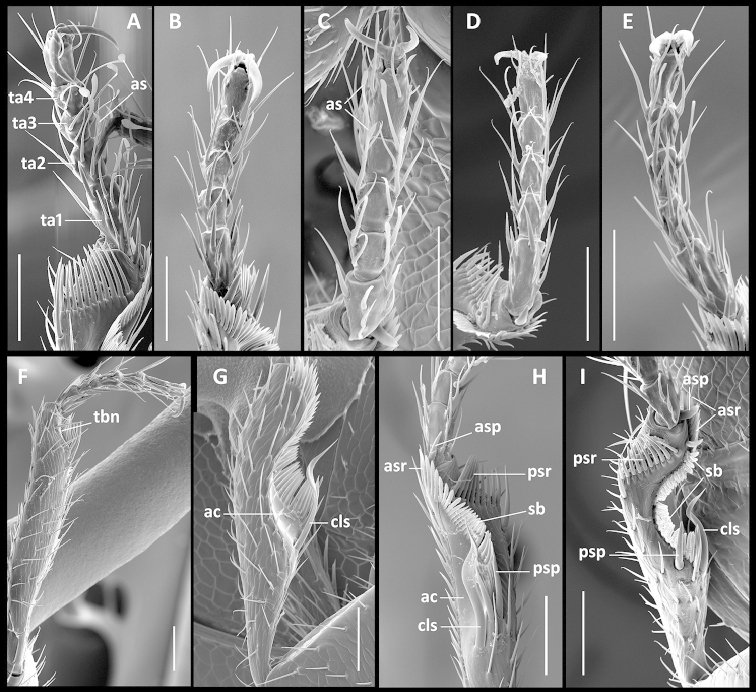
SEM illustrations of structural features of front legs of *Geocharidius* species. **A–E** protarsi, ventral aspect: **A** right protarsus of *Geocharidius jalapensis*, male **B** left protarsus of *Geocharidius jalapensis*, female **C** right protarsus of *Geocharidius erwini*, male **D** right protarsus of *Geocharidius minimus*, female **E** left protarsus of *Geocharidius minimus*, male **F–I** protibia: **F** right protibia of *Geocharidius andersoni*, dorso-lateral aspect **G** left protibia of *Geocharidius lencanus*, lateral aspect **H** left protibia of *Geocharidius comayaguanus*, ventral aspect **I** right protibia of *Geocharidius erwini*, ventral aspect. Legend: ac – antenna cleaner; as – adhesive seta; asp – anterior spur; asr – anterior setal row; cls – clip seta; psp – posterior spur; psr – posterior setal row; sb – setal band; ta1-ta4 – tarsomeres 1-4. Scale = 0.05mm.

**Figures 7.**
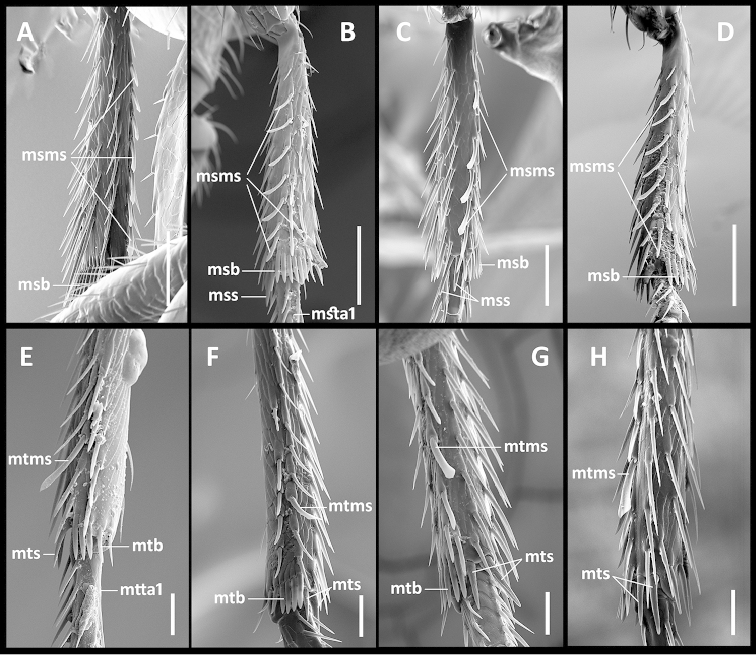
SEM illustrations of structural features of meso- and metatibia of *Geocharidius* species, various aspects. **A–D** mesotibia: **A** right mesotibia of *Geocharidius jalapensis*, ventral aspect **B** left mesotibia of *Geocharidius minimus*, medial aspect **C** left mesotibia of *Geocharidius longinoi*, ventral aspect **D** left mesotibia of *Geocharidius comayaguanus*, medial aspect **E–H** metatibia: **E** right metatibia of *Geocharidius zullinii*, medial aspect **F** left metatibia of *Geocharidius balini*, medial aspect **G** left metatibia of *Geocharidius longinoi*, ventral aspect **H** right metatibia of *Geocharidius comayaguanus*, ventral aspect. Legend: msb – mesotibial brush; msms – mesotibial modified seta; mss – mesotibial spur; msta1 – mesotarsus 1; mtb – metatibial brush; mtms – metatibial modified seta; mts – metatibial spur; mtta1 – metatarsus 1. Scale = 0.02mm.

Legs. Moderate in length, not elongate. Prothoracic legs of males with 1^st^ protarsomere not dilated, but with varied setal pattern. In some species (Fig. 6A) tarsomeres 1-4 of males with one to three pairs of slightly dilated adhesive setae (Stork 1980), which are absent from protarsi of females (Fig. 6B); in males of other species, these setae on tarsomeres 2-4 only (Fig. 6C); and in other species, males have adhesive vestiture similar to females (Fig. 6D–E). Protibiae (Fig. 6F–I) with antenna cleaner of type B (Hlavac 1971), with both anterior (asr) and posterior (psr) apical setal rows and concave apico-lateral notch (tbn). Profemora moderately swollen. Mesotibiae (Fig. 7A–D) with two terminal spurs (mss), tibial brush (msb) and a row of modified setae posteriolaterally (msms). Metafemora unmodified, metatibiae (Fig. 7E–H) with two terminal spurs (mts), tibial brush (mtb) and one modified seta (mtms) in posteriolateral setal row. Tarsi pentamerous, last and 1^st^ tarsomeres are the longest, 2-4 tarsomeres of equal length on the tarsi of all legs, 1^st^ tarsomere shorter than combined length of 2-4 tarsomeres. Tarsal claws simple, untoothed.

Abdominal ventrites. Five visible abdominal ventrites: 2^nd^ ventrite longest, more than 3 times longer than 3^rd^ or 4^th^, 3^rd^ and 4^th^ equal in length; the last, 5^th^, approximately 1.5 times longer than 4^th^. Intercoxal process (ipa) of 2^nd^ ventrite broad (Fig. 3D–F), widely triangular.

Male genitalia. Median lobe of aedeagus anopic, elongate, arcuate and twisted. Internal sac with dorsal copulatory sclerites only, which are long, longer than half of length of the median lobe, except in *Geocharidius comayaguanus* male in which they are rather short (Fig. 19K–L, O–P). Sclerites in form of a long fig, rounded or pointed at basal end, and typically tapered into a long flagellum in apical half, in a few species tapered as a short blade-like structure. Additional spines of internal sac absent. Parameres bisetose. Left paramere large and relatively narrow, mostly with long and narrow apical constriction. Right paramere small and narrow. Ring sclerite (Figs 10, 16, 20) ovate or triangular-ovate with an elongate handle-like extension of varied shape.

Ovipositor. Gonocoxite 1 asetose. Gonocoxite 2 triangular, 1.8–2.0 times longer than its basal width, slightly to moderately curved, with lateral and medial ensiform and two apical nematiform setae. Ensiform setae of similar length and shape. Laterotergite with 5-7 setae.

Female internal genitalia. Spermatheca simple (Figs 11, 17, 21), not bilobate, either unsclerotized fusiform or sclerotized of varied shape, typically fusiform with a bulb-like enlargement apically, straight or bent rectangularly. Parts of spermatheca mostly undifferentiated and named in relation to point of attachment of the spermatecal gland: cornu from point of gland attachment to the apex, and nodulus from point of gland attachment to point of duct attachment (Fig. 11). The ramus, the protruding area of attachment of the spermathecal gland (Maddison 1993), is flat and not developed in the species under consideration here.

**Included taxa.** The *integripennis* species group includes three previously described species: *Geocharidius integripennis* (Bates), *Geocharidius zullinii* Vigna Taglianti, *Geocharidius gimlii* Erwin, and twelve new species, described below: *Geocharidius andersoni*, sp. n., *Geocharidius vignatagliantii*, sp. n., *Geocharidius erwini*, sp. n., *Geocharidius minimus*, sp. n., *Geocharidius jalapensis*, sp. n., *Geocharidius balini*, sp. n., *Geocharidius longinoi*, sp. n., *Geocharidius antigua*, sp. n., *Geocharidius lencanus*, sp. n., *Geocharidius celaquensis*, sp. n., *Geocharidius comayaguanus*, sp. n. and *Geocharidius disjunctus*, sp. n.

**Geographical distribution.** The species of this group now are known from mountain ranges of southern Mexico (state of Chiapas), Guatemala and Honduras (Fig. 22).

**Way of life.** According to label information, specimens of this group were collected from leaf litter within the 2050–2950 m elevation range in the mountains of southern Mexico, within the 1600–3200 m range in the mountains of Guatemala, and within the 1300–2500 m range in the mountains of Honduras. Beetles were extracted from litter in oak, pine, pine-oak, mixed hardwood (without oaks), cloud and lower and upper montane forests. Months of collection include May through September and November.

**Relationships.** The position of the *integripennis* group species within the genus is unclear at present and awaits further morphological study of the globose representatives of *Geocharidius* and a molecular phylogenetic analysis of all species.

#### A key for identification of adults of the *integripennis* species group of *Geocharidius* Jeannel

The following key includes all known members of the *integripennis* species group. The key makes use of distributional information because each of the three countries mentioned has its own *Geocharidius* assemblage, the ranges of which are non-overlapping with those of neighboring countries. Our current information on species distributions may prove to be incomplete with additional sampling, so dissection and examination of genitalia should be used for confirmation wherever possible.

**Table:** 

1	Body form slightly to moderately convex (Fig. 2A–C) and EITHER with head (Fig. 1A–C) and elytra (Fig. 1G–H) totally covered with microsculpture throughout OR, if only head covered with microsculpture, then elytra subparallel with prominent rounded humeri (Fig. 8C)	**2**
–	Body forms globose, with markedly convex elytra (Fig. 2D) and EITHER frontal area of head without microsculpture OR, if frontal area with microsculpture, then elytra ovoid with widely rounded humeri (Fig. 78, p. 76, Sokolov 2013)	**other groups of *Geocharidius***
2	Specimen from Mexico	**3**
–	Specimen from Guatemala	**5**
–	Specimen from Honduras	**12**
3	Specimen larger (SBL range 1.40–1.61 mm). Habitus as in Fig. 8C. Elytra almost subparallel in basal two-thirds with rectangularly rounded humeri. Microsculpture developed only on head; pronotum and elytra smooth, without evident microscuplture	***Geocharidius andersoni* sp. n.**
–	Specimen smaller (SBL range 1.18–1.40 mm). Habitus as in Fig. 8A–B. Elytra subparallel only in middle part, humeri broadly rounded. Microsculpture present on head and elytra, only pronotum smooth	**4**
4	Specimen from the Chiapas Highlands. Male with shaft of median lobe of male aedeagus (Fig. 9A, D) widened apically and with dorsal sclerites of internal sac compact. Female with spermatheca unsclerotized, long and fusiform (Fig. 11A)	***Geocharidius zullinii* Vigna Taglianti**
–	Specimen from the Sierra Madre de Chiapas. Male with shaft of median lobe of aedeagus (Fig. 9E) subparallel and with dorsal sclerites of internal sac partitioned. Female with spermatheca sclerotized, short and bean-like (Fig. 11B)	***Geocharidius vignatagliantii* sp. n.**
5	Elongate (Fig. 12H), smaller on average (SBL range 1.11–1.24 mm). Pronotum with basal margin narrower (WPa/WPp 1.06±0.024). Pronotum and proepisternum smooth. Male with dorsal sclerites of internal sac in form of a flagellum with basal part slightly widened and bent laterally (Fig. 13G, H). Female with spermatheca as in Fig. 17C	***Geocharidius minimus* sp. n.**
–	Elongate ovoid (Fig. 12A–G), larger on average (SBL range 1.16–1.57 mm). Pronotum with basal margin wider (WPa/WPp<1.03). If pronotum smooth, then proepisternum with microsculpture. If male with dorsal sclerites of internal sac formed as a long flagellum (Fig. 19A–B), then their base not bent laterally. Spermatheca of female, if fusiform, then with distinct apical bulb-like enlargement (Fig. 17B, D, E)	**6**
6	Pronotum wider basally, width between posterior angles greater than between anterior angles (Wm/Wp<0.97)	**7**
–	Pronotum with narrower basal margin, width between posterior angles equal to that of anterior angles (0.97<Wm/Wp<1.03)	**8**
7	Specimen smaller (SBL range 1.26–1.29 mm). Habitus as in Fig. 12G. Male with dorsal sclerites of internal sac formed as a flagellum-like structure apically, abruptly and markedly widened towards rounded basal part (Fig. 19A–B). Female with spermatheca short (Fig. 21A), with swollen nodulus. Specimen from the volcanic chain of the Guatemalan Cordillera	***Geocharidius antigua* sp. n.**
–	Specimen larger (SBL range 1.34–1.51 mm). Habitus as in Fig. 12C. Male with dorsal sclerites of internal sac formed as a wavy, ribbon-like structure (Fig. 13R), of approximately equal width along its entire length. Female with spermatheca elongate, of similar breadth along entire length (Fig. 17F). Specimen from the interior: the Sierra de las Minas range	***Geocharidius longinoi* sp. n.**
8	Pronotum smooth (as in Fig. 1E). Male with dorsal sclerites of internal sac markedly extended basally through basal orifice (Fig. 13A, D) as a long and narrow fig, widened and rounded basally. Female with spermatheca, if fusiform, then also curved (Fig. 17B)	**9**
–	Pronotum covered with microsculpture (Fig. 1D). Male with dorsal sclerites of internal sac slightly extended basally through basal orifice, EITHER elongate and pointed basally (Figs 13K–L) OR formed as a wide fig (Fig. 13O). Female with spermatheca fusiform and straight, not bent (Fig. 17D–E)	**11**
9.	Specimen smaller on average (SBL range 1.16–1.31 mm). Habitus as in Fig. 12E. Male with dorsal sclerites of internal sac gradually dilated basally to straight basal part (Fig. 13D). Spermatheca of female fusiform (Fig. 13B)	***Geocharidius erwini* sp. n.**
–	Specimen larger on average (SBL range 1.33–1.43 mm). Habitus as in Fig. 12A–B. Male with dorsal sclerites of internal sac abruptly dilated basally with enlargement curved ventrally (Figs 13A, 15). Female with spermatheca bean-shaped (Fig. 17A)	**10**
10.	Body form broad, ovoid (Fig. 12A). Pronotum markedly transverse (Wm/Le=1.32) and distinctly constricted posteriorly (Wm/Wp= 1.38). Specimen from the Sierra de los Cuchumatanes (Huehuetenango Department)	***Geocharidius gimlii* Erwin**
–	Body form narrow, elongate ovoid (Fig. 12B). Pronotum less transverse (Wm/Le=1.25±0.009) and less constricted toward base (Wm/Wp= 1.33±0.002). Specimen from the Cerro Maria Tecún (Totonicapán Department)	***Geocharidius integripennis* (Bates)**
11.	Specimen smaller on average (SBL range 1.22–1.34 mm). Habitus as in Fig. 12F. Male with dorsal sclerites of internal sac formed as a wide fig widened basally (Fig. 13O). Female with spermathecal gland short (Fig. 17E)	***Geocharidius balini*, sp. n.**
–	Specimen larger on average (SBL range 1.33–1.57 mm). Habitus as in Fig. 12D. Male with dorsal sclerites of internal sac formed as an elongate fig, tapered and pointed basally (Figs 13K–L). Female with spermathecal gland long, longer than spermatheca (Fig. 17D)	***Geocharidius jalapensis* sp. n.**
12.	Pronotum and proepisternum covered with microsculpture. Male with dorsal sclerites of internal sac long, only slightly widened basally (Fig. 19E, H). Female with spermatheca slightly dilated in apical fourth and with point of spermathecal gland attachment closer to the apex (Fig. 21B–C)	**13**
–	Pronotum and proepisternum smooth, without evident microsculpture. Male with dorsal sclerites of internal sac EITHER short (Fig. 19K–L, O–P) OR, if long, then these abruptly and markedly dilated basally (Fig. 19Q, T). Female with point of spermathecal gland attachment to spermatheca closer to the basal end and EITHER tapered apically OR of similar breadth over apical half (Fig. 21D–E)	**14**
13.	Specimen larger (SBL range 1.30–1.47 mm). Habitus as in Fig. 18B. Male with apical part of median lobe of aedeagus attenuated (Fig. 19H). Female with spermatheca curved apically (Fig. 21C)	***Geocharidius lencanus* sp. n.**
–	Specimen smaller (SBL range 1.15–1.20 mm). Habitus as in Fig. 18A. Male with apical part of median lobe of aedeagus of average shape (Fig. 19E). Female with spermatheca straight (Fig. 21B)	***Geocharidius celaquensis* sp. n.**
14.	Male with dorsal sclerites of internal sac short (Figs 19K–L, O–P). Female with spermatheca tapered apically (Fig. 21D)	***Geocharidius comayaguanus* sp. n.**
–	Male with dorsal sclerites of internal sac long (Figs 19Q, T). Spermatheca of female of similar breadth throughout apical half (Fig. 21E)	***Geocharidius disjunctus* sp. n.**

#### Species from Mexico

##### Geocharidius andersoni sp. n.

Taxon classificationAnimaliaColeopteraCarabidae

http://zoobank.org/97465882-51B8-473D-899A-6A0300DDAB82

Figs 1C, F, I , 6F , 8C , 9H–J , 10C , 11C , 22 , 23

###### Type material.

HOLOTYPE, a male, in CMNH, point-mounted, dissected, labeled: \ MEXICO: Chiapas, Cerro Huitepec (Pico), ca. 5km W San Cristobal, 2750m, 15 IX 1991, R. Anderson,#91-101, ex: cloud forest litter \ CMNH \ HOLOTYPE *Geocharidius andersoni* Sokolov and Kavanaugh 2014 [red label] \. PARATYPES: A total of 4 specimens (1 male and 1 female were dissected), deposited in CAS and CMNH, labeled same as holotype except for one female, which has an additional label \ *Anillinus* sp. det. D. Shpeley 1997 \.

###### Type locality.

Mexico, Chiapas, Chiapas Highlands, Cerro Huitepec.

###### Etymology.

The specific epithet is a Latinized eponym in the genitive case, and is based on the surname of Robert S. Anderson, Curator of Entomology at the Canadian Museum of Nature, Ottawa, Canada, the collector of the type series of this species.

###### Recognition.

Adults of this new species are distinguished from those of other members of the *integripennis* species group by the following combination of external characters: size large, elytra wide and smooth (without microsculpture); and males are further distinguished by the size and structure of the median lobe (Figs 9H–J, 10) and the form of the ring sclerite (Fig. 10C).

###### Description.

*Size*. Large for genus (SBL range 1.40–1.61 mm, mean 1.51±0.077 mm, n=5).

**Figures 8.**
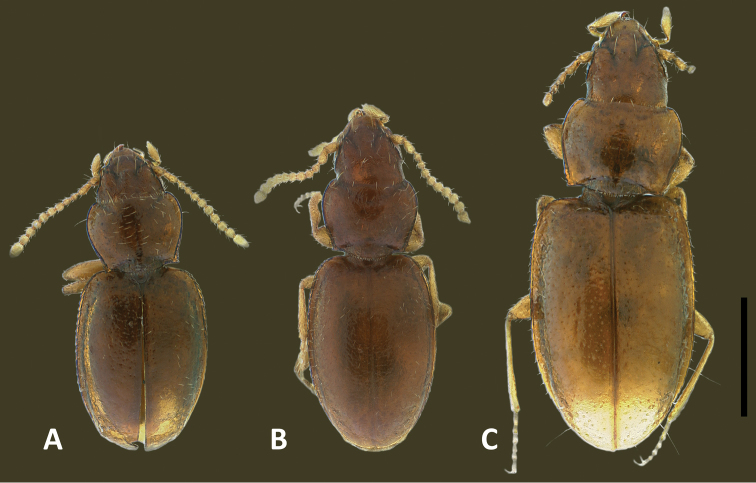
Digital images of habitus, dorsal aspect, of Mexican *Geocharidius* species. **A** *Geocharidius zullinii* (MEXICO, Chiapas, Reserva Huitepec) **B** *Geocharidius vignatagliantii* (MEXICO, Chiapas, Motozintla), paratype **C** *Geocharidius andersoni* (MEXICO, Chiapas, Cerro Huitepec), holotype. Scale = 0.5mm.

Habitus. Body form (Fig. 8C) moderately convex, elongate ovoid, general proportions (WE/SBL 0.42±0.015) wide, head narrow relative to pronotum (WH/WPm 0.69±0.017), pronotum narrow in comparison with elytra (WPm/WE 0.73±0.028).

Color. Body rufotestaceous, appendages testaceous.

Microsculpture. Mesh pattern of irregularly isodiametric sculpticells distinctly present over dorsal surface of head only. Pronotum, elytra and proepisternum smooth (without evident microsculpture).

Head, dorsal aspect (Fig. 1C).

Prothorax. Pronotum (Fig. 1F) moderately transverse (WPm/LP 1.29±0.007), with lateral margins slightly constricted posteriorly (WPm/WPp 1.29±0.020). Posterior angles slightly obtuse (100–110°). Width between posterior angles greater than between anterior angles (WPa/WPp 0.94±0.019).

Elytra (Fig. 8C). Moderately convex, slightly depressed along suture, widest in this species group (WE/LE 0.70±0.025), without traces of striae. Humeri distinct, rounded, in outline forming right angle with longitudinal axis of body. Lateral margins subparallel, slightly divergent at basal fifth, evenly rounded to apex in apical third.

Legs. Protibia (Fig. 6F).

**Figures 9.**
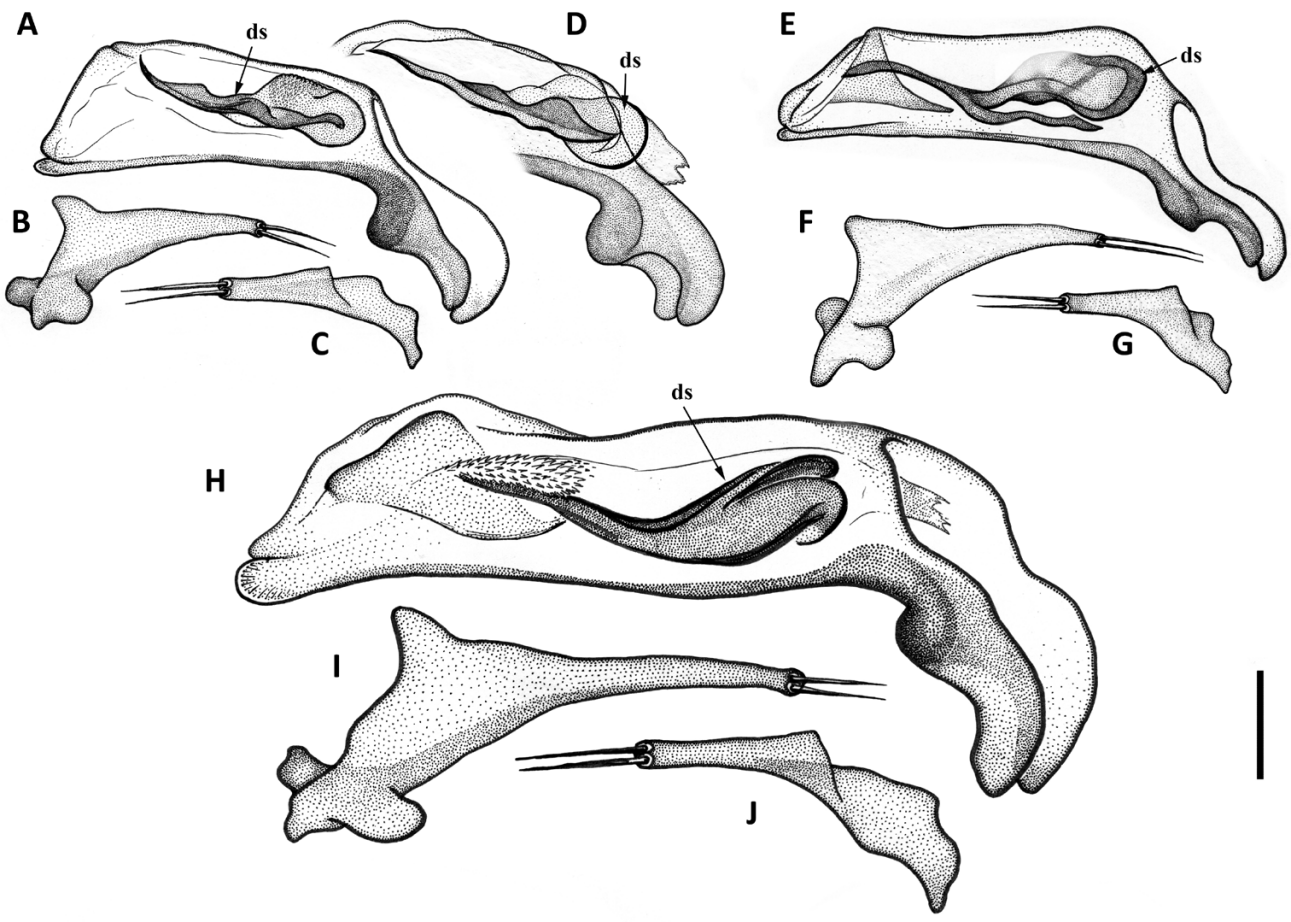
Line drawings of aedeagus of Mexican *Geocharidius* species. *Geocharidius zullinii* (MEXICO, Chiapas, Guadalupe Shankala): **A** median lobe, right lateral aspect **B** left paramere, left lateral aspect **C** right paramere, right lateral aspect. *Geocharidius zullinii* (MEXICO, Chiapas, Reserva Huitepec): **D** part of median lobe, right lateral aspect. *Geocharidius vignatagliantii* (MEXICO, Chiapas, Motozintla), paratype: **E** median lobe, right lateral aspect **F** left paramere, left lateral aspect **G** right paramere, right lateral aspect. *Geocharidius andersoni* (MEXICO, Chiapas, Cerro Huitepec), holotype: **H** median lobe, right lateral aspect **I** left paramere, left lateral aspect **J** right paramere, right lateral aspect. Legend: ds – dorsal sclerites. Scale = 0.05mm.

**Figures 10.**
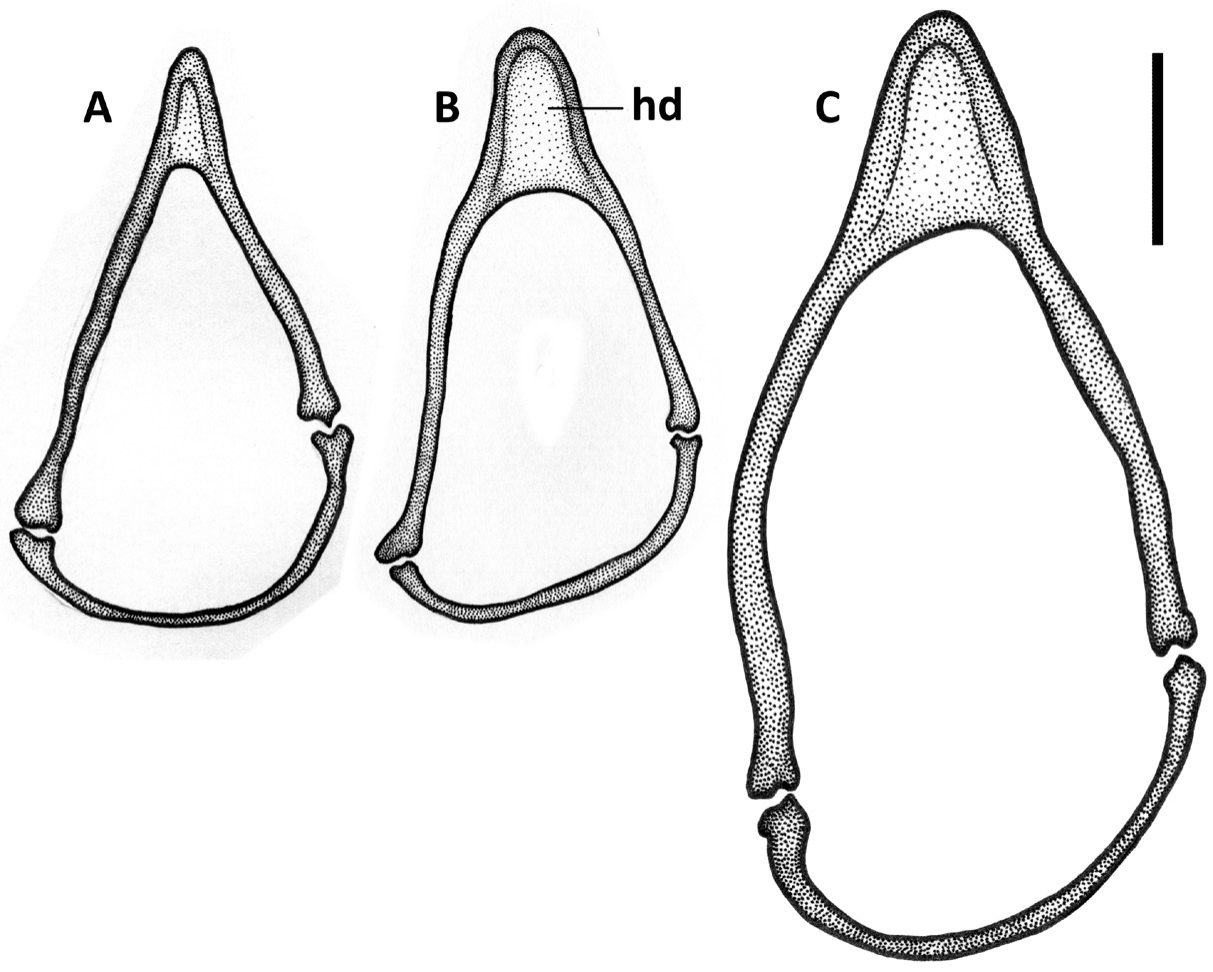
Line drawings of ring sclerite of Mexican *Geocharidius* species, male genitalia, dorsal aspect. **A** *Geocharidius zullinii* (MEXICO, Chiapas, Reserva Huitepec) **B** *Geocharidius vignatagliantii* (MEXICO, Chiapas, Motozintla), paratype **C** *Geocharidius andersoni* (MEXICO, Chiapas, Cerro Huitepec), holotype. Legend: hd – handle of ring sclerite. Scale = 0.1mm.

Male genitalia. Median lobe (Fig. 9H) with very long subparallel shaft, and moderately enlarged apex, broadly rounded at tip. Ventral margin slightly convex medially. Dorsal sclerite of internal sac in form of a long fig, apically tapered into a short flagellum, and gradually widened basally with basal margin bent ventrally. Membranous field near ostium flag with numerous small scales. Right paramere with long and narrow apical constriction (Fig. 9J). Left paramere with very long and narrow apical constriction (Fig. 9I). Ring sclerite with handle triangular, rounded apically (Fig. 10C).

**Figures 11.**
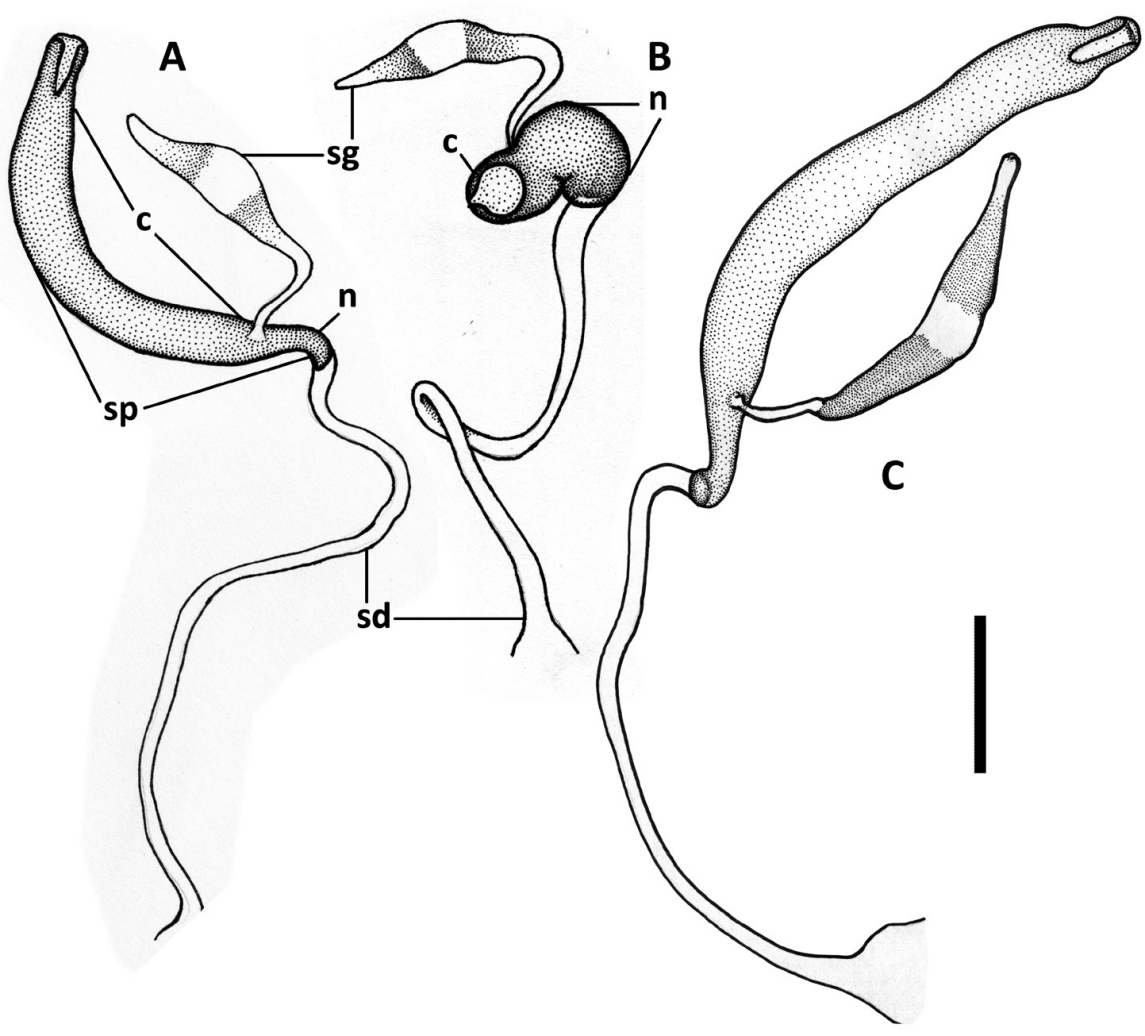
Line drawings of spermatheca of Mexican *Geocharidius* species. **A** *Geocharidius zullinii* (MEXICO, Chiapas, Reserva Huitepec) **B** *Geocharidius vignatagliantii* (MEXICO, Chiapas, Motozintla), paratype **C** *Geocharidius andersoni* (MEXICO, Chiapas, Cerro Huitepec), paratype. Legend: c – cornu; n – nodulus; sd – spermathecal duct; sg – spermathecal gland; sp – spermatheca. Scale = 0.05mm.

Female internal genitalia. Spermatheca unsclerotized, fusiform, arcuate, with cornu long and subparallel and nodulus short and tapered basally (Fig. 11C). Length of spermathecal gland less than length of spermatheca. Spermathecal duct loosely wavy, but not coiled.

###### Geographical distribution.

This species is known only from the type locality in the mountains of the Cerro Huitepec, part of the Chiapas Highlands, State of Chiapas, Mexico (Fig. 22, white diamond).

###### Way of life.

Specimens were extracted from cloud forest litter at an elevation of 2750 m.

###### Relationships.

The shape of the spermatheca of females (Fig. 11C) suggests that this species is closely related to the sympatric *Geocharidius zullinii* Fig. 11A). Dorsal sclerites in the internal sac of *Geocharidius andersoni* males (Fig. 9H) resemble a shortened version of the dorsal sclerites of males of *Geocharidius gimlii* (Fig. 13A), which can be considered as a remote relative.

##### Geocharidius vignatagliantii sp. n.

Taxon classificationAnimaliaColeopteraCarabidae

http://zoobank.org/B1AB6E98-4312-4F87-8B7D-48EE76DB2C82

Figs 8B , 9E–G , 10B , 11B , 22

###### Type material.

HOLOTYPE, a male, in CMNC, point-mounted, labeled: \ MEXICO: Chiapas: Mpio: Motozintla, Benito Juarez, 2050m, 15°22.1'00"N, 92°19'07"W, 28.VII.2005, R. Anderson, oak/pine forest litter 2005-013C \ CMNC \ HOLOTYPE *Geocharidius vignatagliantii* Sokolov and Kavanaugh 2014 [red label] \. PARATYPES: A total of 14 specimens (2 males and 4 females were dissected), deposited in CAS, CMNC and KUNHM; 3 specimens labeled same as holotype; 4 specimens labeled: \ MEXICO: Chiapas: Mpio: Motozintla, Benito Juarez, 2050m, 15°22.1'00"N, 92°19'07"W, 28.VII.2005, R. Anderson, oak/pine forest litter 2005-013A \ CMNC \; 7 specimens labeled: \ MEXICO: Chiapas: Mpio, Motozintla, Benito Juarez 15°22.017'N, 92°19.117'W, 2050m, 28.VII.2005, R. Anderson, oak/pine forest litter MEX 1A05-013 \ SM0711461 KUNHM-ENT \.

###### Type locality.

Mexico, Chiapas, Motozintla, Sierra Madre de Chiapas, Benito Juárez.

###### Etymology.

The specific epithet is a Latinized eponym in the genitive case, and is based on the surname of Prof. Augusto Vigna Taglianti, Director of the Museum of Zoology at the Sapienza University of Rome, Roma, Italia, the first reviser of the species of *Geocharidius*.

###### Recognition.

Adults of this new species are practically indistinguishable externally from those of *Geocharidius zullinii* but can be distinguished from the latter and from the other members of the *integripennis* species group by the structure of the median lobe of males and the shape of spermatheca of females.

###### Description.

Size. Medium for genus (SBL range 1.27–1.40 mm, mean 1.32±0.038 mm, n=5).

Habitus. Body form (Fig. 8B) moderately convex, elongate ovoid, general proportions (WE/SBL 0.40±0.008), proportions of head (WH/WPm 0.73±0.014) and pronotum (WPm/WE 0.75±0.016) average for group.

Color. Body brunneorufous, appendages testaceous.

Microsculpture. Present over all dorsal surfaces of head and elytra. Pronotum and proepisternum smooth.

Prothorax. Pronotum moderately transverse (WPm/LP 1.26±0.021), with lateral margins moderately constricted posteriorly (WPm/WPp 1.32±0.020). Posterior angles obtuse (110–120°). Widths between anterior and posterior angles of equal length (WPa/WPp 0.99±0.020).

Elytra. Moderately convex, slightly depressed along suture, moderately wide (WE/LE 0.68±0.016), without traces of striae. Humeri broadly rounded, in outline forming right angle with longitudinal axis of body. Lateral margins convex, evenly divergent at basal third, evenly rounded to apex in apical third.

Male genitalia. Median lobe of aedeagus (Fig. 9E) with long subparallel shaft, and small rounded apex. Ventral margin almost straight. Dorsal sclerites of internal sac in form of a long fig, apically tapered into a rather long flagellum, and abruptly widened basally as a semicircular end fig near basal orifice. Right paramere with long and narrow apical constriction (Fig. 9G). Left paramere with long and narrow apical constriction (Fig. 9F). Ring sclerite with handle triangular, widely rounded apically (Fig. 10B).

Female internal genitalia. Spermatheca sclerotized, bean-shaped, arcuate, with cornu short and nodulus long (Fig. 11B). Length of spermathecal gland greater than length of spermatheca. Spermathecal duct not coiled.

###### Geographical distribution.

This species is known only from the type locality in the mountains of the Sierra Madre de Chiapas, located in the municipality of Motozintla, State of Chiapas, Mexico (Fig. 22, white squares).

###### Way of life.

Specimens were collected by sifting oak/pine forest litter at an elevation of 2050 m.

###### Relationships.

Adults of this species closely resemble those of *Geocharidius zullinii* from the Chiapas Highlands externally and in the shape of dorsal sclerites of the internal sac (Fig. 9E; cf. Fig. 9A). The shape of the spermatheca of females (Fig. 11B) suggests a relationship with the Guatemalan *Geocharidius integripennis* (Fig. 17A) from the Sierra de los Cuchumatanes of the Guatemalan Cordillera.

##### Geocharidius zullinii

Taxon classificationAnimaliaColeopteraCarabidae

Vigna Taglianti

Figs 1B, E, H , 3A , 4A–B, E–F , 5D , 7E , 8A , 9A–D , 10A , 11A , 22 , 23

###### Holotype.

A male, dissected, deposited in A. Vigna Taglianti’s private collection [not examined]. Type locality: Mexico, Chiapas, Chiapas Highlands, Comitàn, S of Agostin (Fig. 22, white circle with dot).

###### Recognition.

Adults of this species (Fig. 8A) are practically indistinguishable from the adults of *Geocharidius vignatagliantii*, described below, and are distinguished from the latter and from the other members of the *integripennis* species group by structure of the median lobe and shape of spermatheca.

###### Description.

The original description provides a thorough accounting of external features of this species and is absolutely sufficient for species characterization. Below, we add references to illustrations of structural features presented here and descriptions of genitalia, which, for females, has not been done previously.

Head, dorsal aspect (Fig. 1B).

Mouthparts. Mandibles (Figs 4A–B, and 4E–F). Maxillae and labium (Fig. 5D).

Prothorax. Ventral aspect (Fig. 3A). Pronotum, lateral margin (Fig. 1E).

Elytra. Lateral margin (Fig. 1H).

Legs. Metatibia (Fig. 7E).

Male genitalia. Median lobe (Fig. 9A) with shaft long, slightly widened apically, apex small and rounded. Ventral margin almost straight. Dorsal sclerites of internal sac in form of a long fig, tapered apically to a rather long blade, and abruptly widen basally as a semicircular end fig near basal orifice. Specimens from the northern part of the geographical range demonstrate slightly different shape of dorsal sclerites (Fig. 9D). Right paramere with long and narrow apical constriction (Fig. 9C). Left paramere with moderately long and gradually tapered apical constriction (Fig. 9B). Ring sclerite with handle triangular, narrowly rounded apically (Fig. 10A).

Female internal genitalia. Spermatheca unsclerotized, fusiform, arcuate, with cornu long and subparallel and nodulus short and basally tapered (Fig. 11A). Length of spermathecal gland less than length of spermatheca. Spermathecal duct loosely wavy, but not coiled.

###### Geographical distribution.

This species is widely distributed across the Chiapas Highlands, State of Chiapas, Mexico (Fig. 22, white circles). We have examined a total of 15 specimens (6 males and 3 females dissected) from the following localities: 4 specimens labeled: MEXICO: Chiapas: Mpio, Huixtán, Guadalupe Shankala, 16°38'N, 92° 25'W, 2350m, 25.VII.2005, R. Anderson, mixed hardwood (no oaks) forest litter MEX 1A05-007 \ SM0701883 KUNHM-ENT \; 2 specimens labeled: MEXICO: Chiapas: Reserva Huitepec, 16°44.686'N, 92°41.312'W, 2600m, 9-VII-2007 M.G.Branstetter ex. winkler, cloud forest leaf litter LLAMA07 MGB629 \ SM0781461 KUNHM-ENT \; 3 specimens labeled: \ MEXICO: Chiapas: Reserva Huitepec, 16°44.686'N, 92°41.312'W, 2600m, 11-VII-2007 J.Longino ex. winkler, under pines, cloud forest edge, leaflitter LLAMA07 JTL6037-s \ SM0786461 KUNHM-ENT \; 3 specimens labeled: \ MEXICO: Chiapas: Reserva Huitepec, 16°44.686'N, 92°41.312'W, 2600m, 11-VII-2007 J.Longino ex. winkler, cloud forest, leaflitter LLAMA07 JTL6036-s \ SM0786461 KUNHM-ENT \; 1 specimen labeled: \ MEXICO: Chiapas, Mpio, San Cristobal de las Casas, Reserva Huitepec, 2450m, 16°45.84'N, 92°40.70'W, 2600m, 11-VII-2003, R. Anderson, cloud forest lit., MEX1A03 108 \ SM0477446 KUNHM-ENT \; 1 specimen labeled: \ MEXICO: Chiapas: 15km E San Cristobal 16.74689°N, 92.48985°W, 2500m, 29-V-2008, sifted leaf litter, cloud forest LLAMA08 Wm-A-05-1 \ SM0836667 KUNHM-ENT \; 1 specimen labeled: \ MEXICO: Chiapas: Mpio, Huixtán, Bazóm, 2450m, 16°44'19.0"N, 92°29'18.3"W, 9-VII-2003, R. Anderson, oak forest litter, MEX1A03 107 \ SMO517781 KUNHM-ENT \.

###### Way of life.

Specimens were sifted from litter in a wide range of different forest types (hardwood without oaks, oak, pine and cloud forests) at elevations of 2350–2600 m.

###### Relationships.

The shape of spermatheca (Fig. 11A) of females suggests that this species is closely related to the sympatric *Geocharidius andersoni* (Fig. 11C), described above.

#### Species from Guatemala

##### Geocharidius antigua sp. n.

Taxon classificationAnimaliaColeopteraCarabidae

http://zoobank.org/2A03829D-0D34-4608-A5A6-9D8909EAEDC2

**Figures 12.**
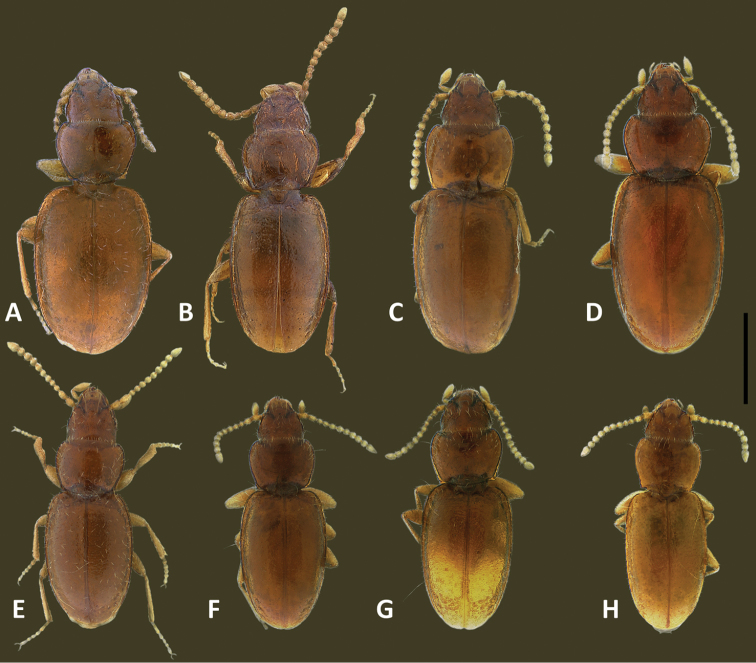
Digital images of habitus of Guatemalan *Geocharidius* species. **A** *Geocharidius gimlii* (GUATEMALA, Huehuetenango, San Juan Ixcoy), holotype **B** *Geocharidius integripennis* (GUATEMALA, Totonicapán, “Totonicapam”), lectotype **C** *Geocharidius longinoi* (GUATEMALA, El Progreso, Cerro Pinalón), paratype **D** *Geocharidius jalapensis* (GUATEMALA, Jalapa, Mataquescuintla), paratype **E** *Geocharidius erwini* (GUATEMALA, Quiché, Los Encuentros), paratype **F** *Geocharidius balini* (GUATEMALA, Suchitepéquez, Volcano Atitlán), paratype **G** *Geocharidius antigua* (GUATEMALA, Sacatepéquez, Antigua), paratype **H** *Geocharidius minimus* (GUATEMALA, Sacatepéquez, Antigua), paratype. Scale = 0.5mm.

Figs 12G , 19A–D , 20A , 21A , 22 , 23

###### Type material.

HOLOTYPE, a male, in KUNHM, point-mounted, dissected, labeled: \ GUATEMALA: Sacatepéquez: 5km SE Antigua, 14.52779 -90.68971±200m, 2350m, 10-VI-2009, ex. sifted leaf litter, oak forest LLAMA09 Wm-B-08-2-all \ KUNHM \ HOLOTYPE *Geocharidius antigua* Sokolov and Kavanaugh 2014 [red label] \. PARATYPES: 1 female, dissected, labeled same as holotype (deposited in KUNHM).

###### Type locality.

Guatemala, Sacatepéquez, 5 km SE of Antigua.

###### Etymology.

The specific epithet is a noun in apposition and refers to the city in the vicinity of which the type series was collected.

###### Recognition.

Adults of this new species are practically indistinguishable in body shape from those of other Guatemalan species of *Geocharidius* with small body size; but the smooth pronotum and presence of microsculpture on the proepisternum form a basis for distinguishing adults of *Geocharidius antigua* from those of sympatric *Geocharidius minimus* and allopatric *Geocharidius balini*, described below. Males and females of *Geocharidius antigua* are distinguished from those of the other members of the *integripennis* species group by the structure of the median lobe and the shape of spermatheca, respectively.

###### Description.

*Size*. Medium for genus (SBL range 1.26–1.29 mm, mean 1.28±0.019 mm, n=2).

Habitus. Body form (Fig. 12G) moderately convex, elongate ovoid, general proportions (WE/SBL 0.39±0.009) and proportions of head (WH/WPm 0.71±0.002) and pronotum (WPm/WE 0.78±0.020) average for group.

Color. Body brunneorufous, appendages testaceous.

Microsculpture. Mesh pattern of irregularly isodiametric sculpticells present over all dorsal surfaces of head and elytra. Pronotum smooth. Proepisternum with evident microsculpture.

Prothorax. Pronotum moderately wide (WPm/LP 1.28±0.011), with lateral margins slightly constricted posteriorly (WPm/WPp 1.29±0.004). Posterior angles slightly obtuse (100–110°). Width between posterior angles greater than between anterior angles (WPa/WPp 0.95±0.022).

Elytra. Moderately convex, slightly depressed along suture, moderately wide (WE/LE 0.67±0.015), without traces of striae. Humeri broadly rounded, in outline forming right angle with longitudinal axis of body. Lateral margins convex, evenly divergent at basal third, evenly rounded to apex in apical third.

Male genitalia. Median lobe of aedeagus (Fig. 19A) with long subparallel shaft, and small rounded apex. Ventral margin almost straight. Dorsal sclerites of internal sac in form of a long fig, tapered apically as a long flagellum, and abruptly widened basally as a nearly circular complex of structures near basal orifice (Figs 19A–B). Right paramere with short and narrow apical constriction (Fig. 19D). Left paramere with long and narrow apical constriction (Fig. 19C). Ring sclerite with handle triangular, slightly asymmetrical, pointed apically (Fig. 20A).

Female internal genitalia. Spermatheca sclerotized, bulb-shaped, straight, and very wide, with cornu short and nodulus swollen (Fig. 21A). Length of spermathecal gland greater than length of spermatheca. Spermathecal duct loosely coiled.

###### Geographical distribution.

This species is known only from the type locality, situated on the northern slopes of volcano Agua in the volcanic chain of the Guatemalan Cordillera (Fig. 22, yellow quadrangle).

###### Way of life.

Specimens were collected by sifting oak forest litter at an elevation of 2350 m.

###### Relationships.

The shape of dorsal sclerites of the internal sac (Fig. 19A–B) suggests that this species is closely related to the Honduran *Geocharidius disjunctus* (Fig. 19Q, T), described below.

**Figures 13.**
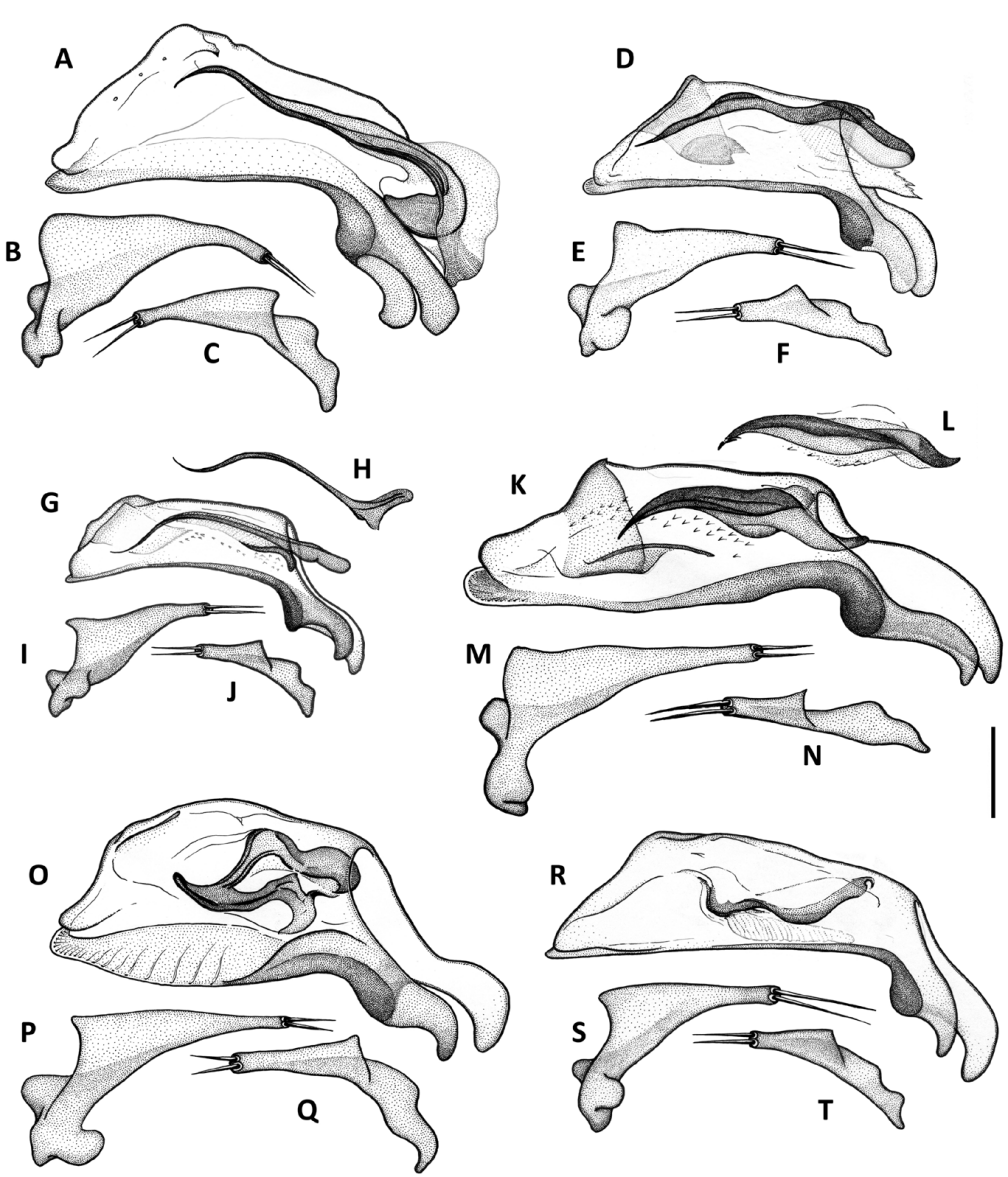
Line drawings of aedeagus of Guatemalan *Geocharidius* species. **A–C** *Geocharidius gimlii* (GUATEMALA, Huehuetenango, San Juan Ixcoy), holotype: **A** median lobe with internal sac and dorsal sclerites, right lateral aspect **B** left paramere, left lateral aspect **C** right paramere, right lateral aspect **D–F** *Geocharidius erwini* (GUATEMALA, Quiché, Los Encuentros), paratype: **D** median lobe with internal sac and dorsal sclerites, right lateral aspect **E** left paramere, left lateral aspect **F** right paramere, right lateral aspect **G–J** *Geocharidius minimus* (GUATEMALA, Sacatepéquez, Antigua), paratype: **G** median lobe with internal sac and dorsal sclerites, right lateral aspect **H** dorsal sclerite of median lobe, dorsal aspect **I** left paramere, left lateral aspect **J** right paramere, right lateral aspect **K–N** *Geocharidius jalapensis* (GUATEMALA, Jalapa, Mataquescuintla), paratype: **K** median lobe with internal sac and dorsal sclerites, right lateral aspect **L** dorsal sclerite of median lobe, dorsal aspect **M** left paramere, left lateral aspect **N** right paramere, right lateral aspect **O–Q** *Geocharidius balini* (GUATEMALA, Suchitepéquez, Volcano Atitlán), paratype: **O** median lobe with internal sac and dorsal sclerites, right lateral aspect **P** left paramere, left lateral aspect **Q** right paramere, right lateral aspect **R–T** *Geocharidius longinoi* (GUATEMALA, El Progreso, Cerro Pinalón), paratype: **R** median lobe with internal sac and dorsal sclerites, right lateral aspect **S** left paramere, left lateral aspect **T** right paramere, right lateral aspect. Scale = 0.05mm.

##### Geocharidius balini sp. n.

Taxon classificationAnimaliaColeopteraCarabidae

http://zoobank.org/4AC8143D-8FDF-49B2-AC46-0AEBC7FE0D32

Figs 1A, D, G , 5B , 7F , 12F , 13O–Q , 16E , 17E , 22 , 23

###### Type material.

HOLOTYPE, a male, in KUNHM, point-mounted, labeled: \ GUATEMALA: Suchitepéquez: 4km S Vol. Atitlán, 14.54915- 91.19055 ±200m, 1625m, 15-VI-2009 ex sifted leaf litter, cloud forest, LLAMA09 Wa-B-09-1-all \ KUNHM \ HOLOTYPE *Geocharidius balini* Sokolov and Kavanaugh 2014 [red label] \. PARATYPES: A total of 117 specimens (3 males and 2 females were dissected), deposited in CAS and KUNHM; 99 specimens labeled same as holotype; 10 specimens labeled: \ GUATEMALA: Suchitepéquez: 4km S Vol. Atitlán, 14.55103- 91.19350 ±306m, 1750m, 15-VI-2009 ex sifted leaf litter, cloud forest, LLAMA09 Wm-B-09-2-01 \ KUNHM \; 7 specimens labeled: \ GUATEMALA: Suchitepéquez: 4km S Vol. Atitlán, 14.55311- 91.19337 ±35m, 1750m, 15-VI-2009 ex sifted leaf litter, cloud forest, LLAMA09 Wm-B-09-2-02 \ KUNHM \; 1 specimen labeled: \ GUATEMALA: Jalapa: 4km E Mataquescuintla, 14.53257 -90.15253 ±200m, 2400m, 1-VI-2009, ex. sifted leaf litter, cloud forest, LLAMA09 Wa-B-07-2-all \ KUNHM \.

###### Type locality.

Guatemala, Suchitepéquez, 4 km S of Volcan Atitlán.

###### Etymology.

The specific epithet is a Latinized eponym in the genitive case, and is based on the given name of the dwarf Balin, a refounder of the underground kingdom of Moria, one of Thorin Oakenshield’s Company of Dwarves who had accompanied Bilbo Baggins on the Quest of Erebor in the book “*The Hobbit, or There and Back Again*” by J.R.R.Tolkien.

###### Recognition.

Adults of this new species are distinguished from those of other members of the *integripennis* species group externally by their small size and the presence of microsculpture on the pronotum and internally by the structure of the median lobe of males and the shape of spermatheca of females.

###### Description.

*Size*. Small to medium for genus (SBL range 1.22–1.34 mm, mean 1.27±0.040 mm, n=26).

*Habitus*. Body form (Fig. 12F) moderately convex, elongate ovoid, general proportions (WE/SBL 0.38±0.008) and proportions of head (WH/WPm 0.72±0.013) and pronotum (WPm/WE 0.78±0.017) average for group.

*Color.* Body brunneorufous, appendages testaceous.

*Microsculpture*. Mesh pattern of irregularly isodiametric sculpticells present over all dorsal surfaces of head, pronotum and elytra. Proepisternum with evident microsculpture.

Head (Fig. 1A).

Mouthparts. Maxillae and labium (Fig. 5B)

Prothorax. Pronotum (Fig. 1D) moderately transverse (WPm/LP 1.26±0.022), with lateral margins moderately constricted posteriorly (WPm/WPp 1.32±0.021). Posterior angles obtuse (110–120°). Widths between anterior and posterior angles of equal length (WPa/WPp 0.99±0.022).

Elytra (Fig. 1G). Moderately convex, slightly depressed along suture, moderately narrow (WE/LE 0.64±0.018), without traces of striae. Humeri rounded, in outline forming right angle with longitudinal axis of body. Lateral margins convex, evenly divergent at basal third, evenly rounded to apex in apical third.

Legs. Metatibia (Fig. 7F)

Male genitalia. Median lobe (Fig. 13O) with shaft short and broad and apex of moderate size and rounded. Ventral margin greatly enlarged and convex, with numerous poriferous canals. Dorsal sclerites of internal sac of peculiar shape, in form of anastomosing short figs, connected in apical and basal thirds, pointed apically as a short blade. Right paramere long and narrow (Fig. 13Q). Left paramere with long and narrow apical constriction (Fig. 13P). Ring sclerite with handle almost rectangularly rounded, slightly asymmetrical (Fig. 16E).

Female internal genitalia. Spermatheca sclerotized, fusiform with apical bulb enlargement, straight, with cornu long and nodulus short (Fig. 17E). Lengths of spermathecal gland and spermatheca equal. Spermathecal duct not coiled.

###### Geographical distribution.

This species is known only from two localities remote from each other: one situated on the southern slopes of volcano Agua in the Suchitepéquez Department, and the other situated on the northern slopes of a former twinned volcano, remains of which form the caldera of Laguna de Ayarza, in Jalapa Department. Both localities are in the volcanic chain of the Guatemalan Cordillera (Fig. 22, green diamonds).

###### Way of life.

Specimens were collected by sifting cloud forest litter at middle (1600–1750 m) to high elevations (2400 m).

###### Relationships.

The shapes of handle of ring sclerite (Fig. 16E) and of the spermatheca (Fig. 17E) suggest that this species is closely related to *Geocharidius jalapensis* (Figs 16D, 17D, described below.

##### Geocharidius erwini sp. n.

Taxon classificationAnimaliaColeopteraCarabidae

http://zoobank.org/6B696BFC-D7AE-4893-BCBC-80579DFAA5B7

Figs 2A , 3B , 5A , 6C, I , 12E , 13D–F , 16B , 17B , 22

Geocharidius integripennis , Sokolov, 2013:53

###### Type material.

HOLOTYPE, a male, in NMNH, glued on cardboard, labeled: \ Guatemala: QUICHÉ, 7km NE Los Encuentros, 2400m, 18.XI.1991 leg. R.Baranowski \ sifting litter under bushes at roadside pine forest \ Loan from USNMNH 2051867 \ HOLOTYPE *Geocharidius erwini* Sokolov and Kavanaugh 2014 [red label] \. PARATYPES: A total of 45 specimens (4 males and 4 females were dissected), deposited in CAS, CMNC, NMNH; 23 specimens labeled same as holotype; 10 specimens labeled: \ Guatemala: QUICHÉ, 7km NE Los Encuentros, 2400m, 14.XI.1991 leg. R.Baranowski \ sifting litter under bushes at roadside pine forest \ Loan from USNMNH 2051867 \; 8 specimens labeled: \ Guatemala QUICHÉ, 6km S Chichicastenango, 2140m. 16.XI.1991 leg. R.Baranowski \ sifting litter, pine-oak forest \ Loan from USNMNH 2051867 \; 4 specimens labeled: \ Guatemala: QUICHÉ, 5km S Chichicastenango, 2000m. 18.XI.1991 leg. R.Baranowski \ sifting litter, pine-oak forest \ Loan from USNMNH 2051867 \; 1 specimen labeled: \ Guat.: QUEZALTEN. 12km S.E. Zunil, Fuentes Georginas, 2460m, 21.VI.1993, R. Anderson, cloud for. litter 93-10A\ CMNC \; 1 specimen labeled: \ Guat.: QUEZALTEN. 12km S.E. Zunil, Fuentes Georginas, 2460m, 21.VI.1993, R. Anderson, cloud for. litter 93-10E\ CMNC \; 1 specimen labeled: \ Guat.: QUEZALTEN. 12km S.E. Zunil, Fuentes Georginas, 2450m, 19.VI.1993, R. Anderson, cloud for. litter 93-5CC \ CMNC \; 1 specimen labeled: \ Guat.: QUEZALTEN. 12km S.E. Zunil, N.W.face Cerro Zunil, 2700m, 20.VI.1993, R. Anderson, hardwood for. litter 93-8F \ CMNC \; 1 specimenslabeled: \ Guat.: QUEZALTENANGO: 12km SE Zunil, NW face Cerro Zunil, hardwd.for.litter, 2700–2760m, R. Anderson 91-30 28.V.1991. \ CMNC \.

###### Type locality.

Guatemala, Quiché Department, 7 km NE of Los Encuentros.

###### Etymology.

The specific epithet is a Latinized eponym in the genitive case and is based on the surname of Terry L. Erwin, Curator of Entomology at the Smithsonian Institution, United States National Museum of Natural History, Washington, D. C., U.S.A., the first reviser of the Guatemalan Anillina.

###### Recognition.

Adults of this new species are practically indistinguishable from those of other the Guatemalan species of *Geocharidius* with small body size. Males and females of *Geocharidius erwini* are distinguished from those of the other members of the *integripennis* species group by the structure of the median lobe of males and the shape of spermatheca of females, respectively.

###### Description.

Size. Small to medium for genus (SBL range 1.16–1.31 mm, mean 1.23±0.057 mm, n=30).

Habitus. Body form (Fig. 12E) moderately convex, elongate ovoid, general proportions (WE/SBL 0.38±0.009) and proportions of head (WH/WPm 0.72±0.012) and pronotum (WPm/WE 0.79±0.015) average for group.

Color. Body rufotestaceous, appendages testaceous.

Microsculpture. Mesh pattern of irregularly isodiametric sculpticells present over all dorsal surfaces of head and elytra. Pronotum smooth. Proepisternum with evident microsculpture.

Mouthparts. Labium (Fig. 5A).

Prothorax. Pronotum moderately transverse (WPm/LP 1.26±0.019), with lateral margins slightly constricted posteriorly (WPm/WPp 1.31±0.025). Posterior angles slightly obtuse (100–110°). Widths between anterior and posterior angles of equal length (WPa/WPp 1.01±0.025). Ventral aspect (Fig. 3B).

Elytra (Fig. 2A). Moderately convex, slightly depressed along suture, moderately wide (WE/LE 0.67±0.017), without traces of striae. Humeri rounded, in outline forming right angle with longitudinal axis of body. Lateral margins convex, evenly divergent at basal third, evenly rounded to apex in apical third.

Legs. Protibia (Fig. 6I). Protarsus (Fig. 6C).

Male genitalia. Median lobe (Fig. 13D) with shaft moderately long, slightly widened apically, and apex small and rounded. Ventral margin almost straight. Dorsal sclerites of internal sac (Fig. 13D) in form of a long fig, tapered apically as a long flagellum, and gradually widen towards semicircular basal end extended basally through the basal orifice. Right paramere with short and wide apical consriction (Fig. 13F). Left paramere with long and moderately narrow apical constriction (Fig. 13E). Ring sclerite with almost rectangularly rounded, subparallel, handle (Fig. 16B).

Female internal genitalia. Spermatheca sclerotized, fusiform with bulb enlargement apically, twice bent rectangularly in opposite directions, with cornu long and nodulus short (Fig. 17B). Length of spermathecal gland less than length of spermatheca. Spermathecal duct coiled.

###### Geographical distribution.

This species is known from a few scattered localities in the Quiché and Quetzaltenango Departments of Guatemala (Fig. 22, green circles).

###### Way of life.

Specimens were collected by sifting litter from different habitats: cloud, hardwood, pine and pine-oak forests at elevations of 2140-2760 m.

###### Relationships.

The shape of dorsal sclerites of the internal sac in males suggests a remote relationship with *Geocharidius minimus* (Fig. 13G), described below.

**Figures 14.**
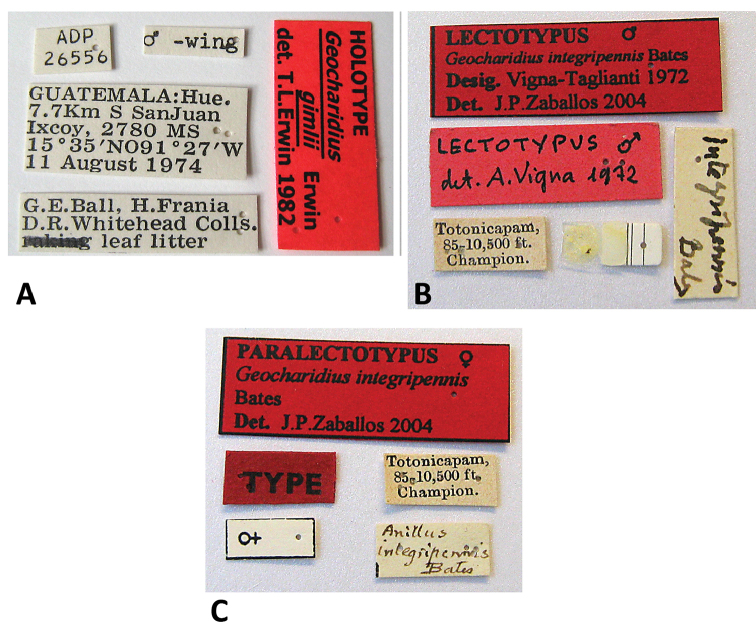
Photographs of labels for type specimens of *Geocharidius* species. **A** *Geocharidius gimlii*, holotype **B** *Geocharidius integripennis*, lectotype **C** *Geocharidius integripennis*, paralectotype.

##### Geocharidius gimlii

Taxon classificationAnimaliaColeopteraCarabidae

Erwin

**Figures 15.**
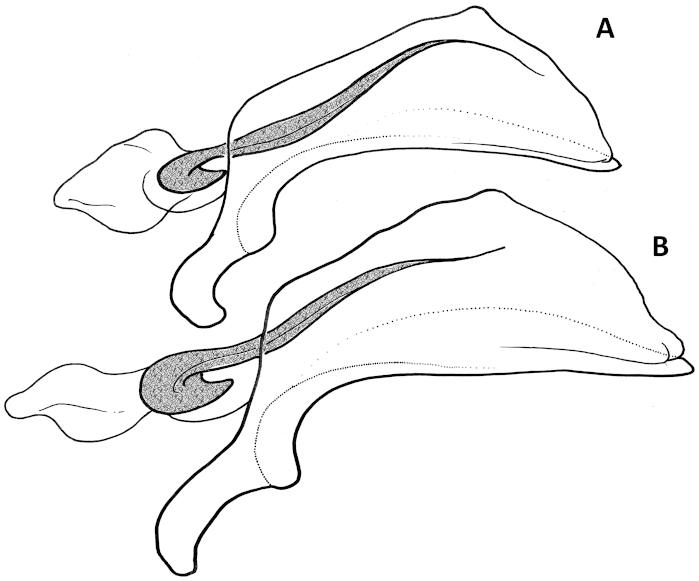
Schematic line drawings of median lobe and dorsal sclerites of internal sac of two previously described Guatemalan *Geocharidius* species. **A** *Geocharidius integripennis* (GUATEMALA, Totonicapán, “Totonicapam”), holotype **B** *Geocharidius gimlii* (GUATEMALA, Huehuetenango, San Juan Ixcoy), holotype, both left lateral aspect.

**Figures 16.**
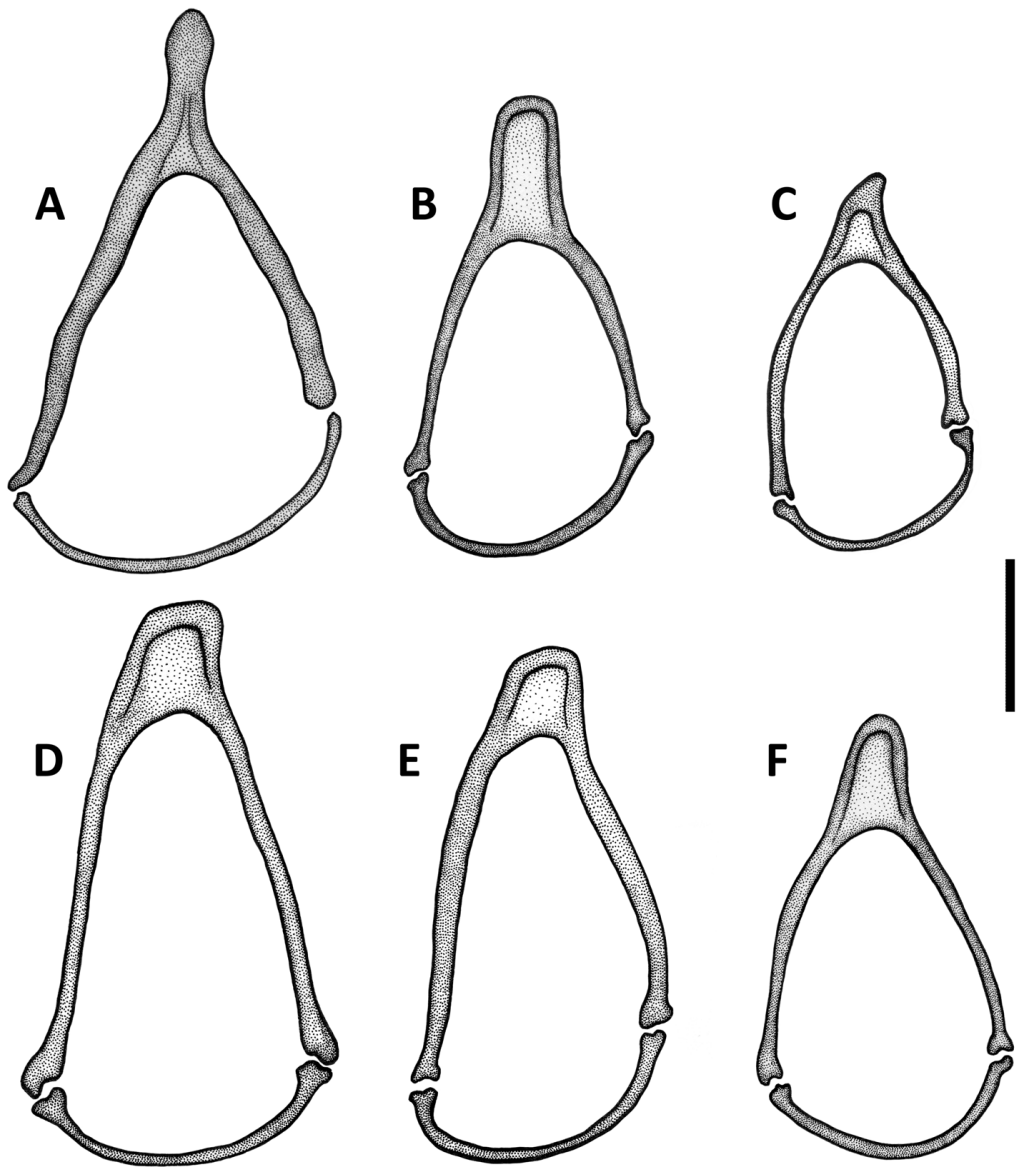
Line drawings of ring sclerite of Guatemalan *Geocharidius* species, male genitalia, dorsal aspect. **A** *Geocharidius gimlii* (GUATEMALA, Huehuetenango, San Juan Ixcoy), holotype **B** *Geocharidius erwini* (GUATEMALA, Quiché, Los Encuentros), paratype **C** *Geocharidius minimus* (GUATEMALA, Sacatepéquez, Antigua), paratype **D** *Geocharidius jalapensis* (GUATEMALA, Jalapa, Mataquescuintla), paratype **E** *Geocharidius balini* (GUATEMALA, Suchitepéquez, Volcano Atitlán), paratype **F** *Geocharidius longinoi* (GUATEMALA, El Progreso, Cerro Pinalón), paratype. Scale = 0.1mm.

Figs 12A , 13A–C , 14A , 15B , 16A , 22

Geocharidius gimlii Erwin, 1982: 488.

###### Holotype.

A male, deposited in NMNH, point-mounted, dissected, labeled (Fig. 14A): \ ♂ –wing \ ADP 26556 \ GUATEMALA: Hue. 7.7km S SanJuan Ixcoy, 2780 MS 15 35'N, 091 27'W 11 August 1974 \ G. E. Ball, H. Frania, D.R. Whitehead Colls. leaf litter \. Type locality. Guatemala, Huehuetenango Department, 7.7 km S of San Juan Ixcoy.

###### Recognition.

Males of this species are distinguished from those of other members of the *integripennis* species group by the following combination of characters: pronotum small, transverse, elytra comparatively wide and structure of median lobe of male as in Fig. 13A.

###### Description.

Size. Medium for genus (SBL 1.42 mm).

Habitus. Body form (Fig. 12A) moderately convex, broadly ovoid; general proportions (WE/SBL 0.41) rather wide; proportions of head (WH/WPm 0.72) average for group; pronotum narrow (WPm/WE 0.74) relative to elytra.

Color. Body rufotestaceous, appendages testaceous.

Microsculpture. Mesh pattern of irregularly isodiametric sculpticells present over all dorsal surfaces of head and elytra. Pronotum smooth (without evident microsculpture). Proepisternum with microsculpture.

Prothorax. Pronotum transverse (WPm/LP 1.32), with lateral margins markedly constricted posteriorly (WPm/WPp 1.38). Posterior angles obtuse (112°). Widths between anterior and posterior angles equal (WPa/WPp 1.01).

Elytra. Moderately convex, slightly depressed along suture, markedly wide (WE/LE 0.70), without traces of striae. Humeri broadly rounded, in outline forming slightly obtuse angle with longitudinal axis of body. Lateral margins convex, evenly divergent at basal half, evenly rounded to apex in apical half.

Male genitalia. Median lobe of aedeagus (Fig. 13A, 15B) with shaft long, widened apically, and apex small and acutely rounded. Ventral margin straight. Dorsal sclerites of internal sac in form of very long fig, protruding from basal orifice, and tapered apically in rather long flagellum, abruptly widened basally as a semicircular dilation, bent ventrally and surrounded by complex of semisclerotized sheaths of peculiar shape. Right paramere with long, narrow apical constriction (Fig. 13C). Left paramere with long, narrow and curved apical constriction (Fig. 13B). Ring sclerite of triangular shape, with sinuations on both sides of long basal handle (Fig. 16A).

Female internal genitalia. Females unknown.

###### Geographical distribution.

This species is known only from the type locality in the mountains of the Sierra de los Cuchumatanes, located in the Huehuetenango Department of Guatemala (Fig. 22, white triangle).

###### Way of life.

The unique type specimen was sifted from leaf litter in Lower Montane Wet Forest (Erwin 1982) at an elevation of 2780 m.

###### Relationships.

The shape of the median lobe in the holotype of *Geocharidius gimlii* (Fig. 15B) is almost identical to that of the male holotype of *Geocharidius integripennis* (Fig. 15A); hence, at least for now, the latter can be considered as its closest relative. The general shape of the dorsal sclerites of the internal sac (namely the apically tapered fig, widened and ventrally bent at the basal end) is also similar to that in *G andersoni* (Fig. 9H) males described above.

##### Geocharidius integripennis

Taxon classificationAnimaliaColeopteraCarabidae

(Bates)

**Figures 17.**
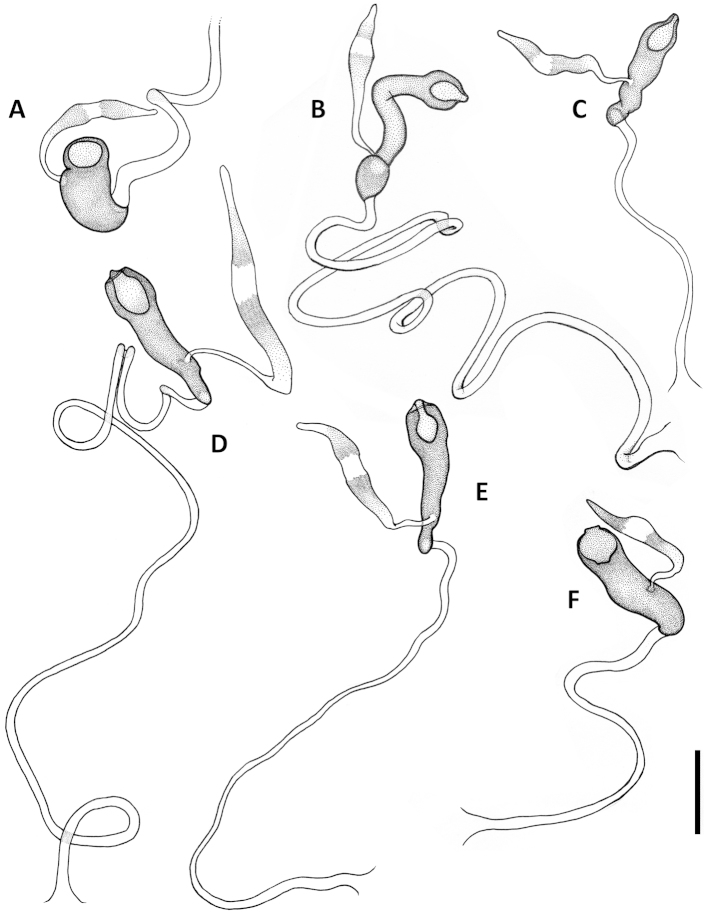
Line drawings of spermatheca of Guatemalan *Geocharidius* species. **A** *Geocharidius integripennis* (GUATEMALA, Totonicapán, “Totonicapam”), holotype **B** *Geocharidius erwini* (GUATEMALA, Quiché, Los Encuentros), paratype **C** *Geocharidius minimus* (GUATEMALA, Sacatepéquez, Antigua), paratype **D** *Geocharidius jalapensis* (GUATEMALA, Jalapa, Mataquescuintla), paratype **E** *Geocharidius balini* (GUATEMALA, Suchitepéquez, Volcano Atitlán), paratype **F** *Geocharidius longinoi* (GUATEMALA, El Progreso, Cerro Pinalón), paratype. Scale = 0.05mm.

Figs 12B , 14B , 14C , 15A , 17A , 22

Anillus integripennis Bates, 1882: 145.

###### Lectotype.

A male, deposited in MNHN, glued on cardboard, dissected, labeled (Fig. 14B): \ Totonicapam, 85- 10,500 ft. Champion \ *integripennis* Bates \ Lectotypus ♂ det. A. Vigna 1972 \ Lectotypus ♂ *Geocharidius integripennis* Bates Desig. Vigna- Taglianti 1972 Det. J.P. Zaballos 2004\. Paralectotype female, also in MNHN, glued on cardboard, dissected, labeled (Fig. 14C): \ Totonicapam, 85- 10,500 ft. Champion \ *Anillus integripennis* Bates \ TYPE \ ♀ \ PARALECTOTYPUS ♀ *Geocharidius integripennis* Bates Det. J.P.Zaballos 2004 \. Type locality: Guatemala, Totonicapán Department, Totonicapam [=Totonicapán].

*Geocharidius tagliantii* Erwin, 1982: 494; synonymized by Zaballos (2004).

###### Recognition.

Males and females of this species are distinguished from those of other members of the *integripennis* species group (except *Geocharidius gimlii*, see Relationships above) by the structure of the median lobe of males and the spermatheca of females. Adults of *Geocharidius gimlii* have proportionately much wider elytra than those of *Geocharidius integripennis*.

###### Description.

Size. Medium for genus (SBL range 1.33–1.43 mm, mean 1.38±0.070 mm, n=2).).

Habitus. Body form (Fig. 12B) moderately convex, elongate ovoid, general proportions (WE/SBL 0.40±0.002), proportions of head (WH/WPm 0.74±0.022) and pronotum (WPm/WE 0.77±0.026) average for group.

Color. Body rufotestaceous, appendages testaceous.

Microsculpture. Mesh pattern of irregularly isodiametric sculpticells present over all dorsal surfaces of head and elytra. Pronotum smooth. Proepisternum with microsculpture.

Prothorax. Pronotum slightly transverse (WPm/LP 1.25±0.009), with lateral margins moderately constricted posteriorly (WPm/WPp 1.33±0.002). Posterior angles slightly obtuse (100-110°). Widths between anterior and posterior angles of equal length (WPa/WPp 0.99±0.018).

Elytra. Moderately convex, slightly depressed along suture, moderately wide (WE/LE 0.67±0.005), without traces of striae. Humeri rounded, in outline forming right angle with longitudinal axis of body. Lateral margins convex, evenly divergent at basal fourth, evenly rounded to apex in apical third.

Male genitalia. Male genitalia of the lectotype are mounted in old gum, covered now with a network of numerous cracks, making the objects inside hard to see. Hence, we could examine only general shape of the median lobe and could not discern details of the inner sac or of the parameres or the round sclerite. Based on what we could see, the median lobe of the aedeagus (Fig. 15A) is very similar to that of the *Geocharidius gimlii* holotype.

Female internal genitalia. Spermatheca of the paralectotype sclerotized, bean-shaped, with apical constriction, almost straight, cornu short and nodulus long (Fig. 17A). Length of spermathecal gland greater than length of spermatheca. Spermathecal duct loosely coiled.

###### Geographical distribution.

Precise locality at which the type series of this species was collected is unknown. Presumably, the material that was collected by Champion and served as the basis for the Bates’ description came from the Cerro Maria Tecún mountains in the Totonicapán Department of Guatemala (Fig. 22, black and white triangle), as was shown by Ball and Roughley (1982) for *Pterostichus (Percolaus) championi* (Bates), the locality label of which is identical with that of the *Geocharidius integripennis* type specimens.

###### Way of life.

The type specimens were collected at an elevation of “10,500 ft.” (= 3200 m).

###### Relationships.

Without doubt, the closest relative of *Geocharidius integripennis* is *Geocharidius gimlii*. In view of the similarity in the shape of the median lobes (Fig. 15B; cf. Fig. 15A) of their males and the range of variation of the median lobes seen among other species of the group, it may seem reasonable to consider these taxa as two subspecies of a single species. However, in the absence of sufficient material for more thorough investigation of variation of the external features and structure of the genitalia, we prefer to preserve the “status quo” and consider *Geocharidius gimlii* and *Geocharidius integripennis* as close, but separate species.

##### Geocharidius jalapensis sp. n.

Taxon classificationAnimaliaColeopteraCarabidae

http://zoobank.org/BF792073-053E-4FA8-BA99-759634A24AC7

Figs 2B , 3D, H–I , 4C–D, G–H , 6A–B , 7A , 12D , 13K–N , 16D , 17D , 22 , 23

###### Type material.

HOLOTYPE, a male, in KUNHM, point-mounted, labeled: \ GUATEMALA: Jalapa: 4km E Mataquescuintla, 14.52943 -90.14775 ±105m, 2620m, 2-VI-2009, ex. sifted leaf litter, cloud forest, LLAMA09 Wm-B-07-1-06 \ KUNHM \ HOLOTYPE *Geocharidius jalapensis* Sokolov and Kavanaugh 2014 [red label] \. PARATYPES: A total of 78 specimens (4 males and 4 females were dissected), deposited in CAS and KUNHM; 10 specimens labeled same as holotype; 2 specimens labeled: \ GUATEMALA: Jalapa: 4km E Mataquescuintla, 14.53409 -90.15290 ±28m, 2325m, 3-VI-2009, ex. sifted leaf litter, cloud forest, LLAMA09 Wm-B-07-1-10 \ KUNHM \; 1 specimen labeled: \ GUATEMALA: Jalapa: 4km E Mataquescuintla, 14.52950 -90.14802 ±254m, 2600m, 1-VI-2009, ex. sifted leaf litter, cloud forest, LLAMA09 Wm-B-07-1-01 \ KUNHM \; 20 specimens labeled: \ GUATEMALA: Jalapa: 4km E Mataquescuintla, 14.53257 -90.15253 ±200m, 2400m, 1-VI-2009, ex. sifted leaf litter, cloud forest, LLAMA09 Wa-B-07-2-all \ KUNHM \; 23 specimens labeled: \ GUATEMALA: Jalapa: 4km E Mataquescuintla, 14.52780 -90.14671 ±105m, 2655m, 2-VI-2009, ex. sifted leaf litter, cloud forest, LLAMA09 Wm-B-07-1-04 \ KUNHM \; 16 specimens labeled: \ GUATEMALA: Jalapa: 4km E Mataquescuintla, 14.52987 -90.14908 ±200m, 2600m, 1-VI-2009, ex. sifted leaf litter, cloud forest on ridge top, LLAMA09 Wa-B-07-1-all \ KUNHM \; 6 specimens labeled: \ GUATEMALA: Jalapa: 4km E Mataquescuintla, 14.52705 -90.14671 ±105m, 2660m, 2-VI-2009, ex. sifted leaf litter, cloud forest, LLAMA09 Wm-B-07-1-05 \ KUNHM \.

###### Type locality.

Guatemala, Jalapa Department, 4 km E of Mataquescuintla.

###### Etymology.

The specific epithet is a Latinized adjective in the masculine form based on the name Jalapa, the Department of Guatemala in which the type series was collected.

###### Recognition.

Adults of this new species are distinguished from those of other members of the *integripennis* species group by the following combination of external characters: size large and pronotum transverse and fully covered with microsculpture. Males and female of *Geocharidius jalapensis* are distinguished from those of other members of the *integripennis* species group by the structure of the median lobe and the shape of spermatheca, respectively.

###### Description.

Size. Medium to large for genus (SBL range 1.33–1.57 mm, mean 1.46±0.081mm, n=25).

Habitus. Body form (Fig. 12D) moderately convex, elongate ovoid, general proportions (WE/SBL 0.39±0.007), proportions of head (WH/WPm 0.71±0.019) and pronotum (WPm/WE 0.76±0.021) average for group.

Color. Body brunneorufous, appendages testaceous.

Microsculpture. Mesh pattern of irregularly isodiametric sculpticells present over all dorsal surfaces of head, pronotum and elytra. Proepisternum with evident microsculpture.

Mouthparts. Mandibles (Figs 4C–D, G–H).

Prothorax. Pronotum markedly transverse (WPm/LP 1.30±0.026), with lateral margins moderately constricted posteriorly (WPm/WPp 1.33±0.029). Posterior angles obtuse (110–120°). Widths between anterior and posterior angles of equal length (WPa/WPp 0.99±0.023).

Pterothorax (Fig. 3D).

Elytra (Fig. 2B). Moderately convex, slightly depressed along suture, moderately wide (WE/LE 0.65±0.009), without traces of striae. Humeri rounded, in outline forming right angle with longitudinal axis of body. Lateral margins convex, evenly divergent at basal third, evenly rounded to apex in apical third.

Legs. Mesotibia (Fig. 7A). Protarsus (Fig. 6A–B).

Abdomen. Ventrites 3-5 (Fig. 3H–I).

Male genitalia. Median lobe (Fig. 13K) with shaft long and apex slightly enlarged and rounded. Ventral margin almost straight. Dorsal sclerites of internal sac in form of a long fig, tapered apically and basally in short extensions (Fig. 13K–L). Right paramere with short and rather wide apical constriction (Fig. 13N). Left paramere with long and narrow apical constriction (Fig. 13M). Ring sclerite with almost rectangularly rounded, slightly asymmetrical, handle (Fig. 16D).

Female internal genitalia. Spermatheca sclerotized, fusiform with apical bulb enlargement, straight, with long cornu and short nodulus (Fig. 17D). Length of spermathecal gland greater than length of spermatheca. Spermathecal duct coiled.

###### Geographical distribution.

This species is known only from type locality, situated on the northern slopes of the former twinned volcano, remains of which form the caldera now filled with the waters of Laguna de Ayarza (Jalapa Department). Physiographically, the region is part of the volcanic chain of the Guatemalan Cordillera (Fig. 22, green triangle).

###### Way of life.

Specimens were collected by sifting cloud forest litter at elevations of 2325–2620 m.

###### Relationships.

The shapes of handle of the ring sclerite (Fig. 16D) and of the spermatheca (Fig. 17D) suggest that this species is closely related to *Geocharidius balini* (Figs 13O and 17E), described above.

##### Geocharidius longinoi sp. n.

Taxon classificationAnimaliaColeopteraCarabidae

http://zoobank.org/2037D9B1-5260-4836-8ED4-0D0AE950111F

Figs 5E , 7C, G , 12C , 13R–T , 16F , 17F , 22

###### Type material.

HOLOTYPE, a male, in KUNHM, point-mounted, labeled: \ GUATEMALA: El Progreso: Cerro Pinalón, 15.08392-89.93013 ±55m, 2750m, 1-V-2009 ex. sifted leaf litter, cloud forest, LLAMA09 Wm-B-01-1-04 \ KUNHM \ HOLOTYPE *Geocharidius longinoi* Sokolov and Kavanaugh 2014 [red label] \. PARATYPES: A total of 13 specimens (2 males and 1 female were dissected), deposited in CAS, CMNC and KUNHM; 5 specimens labeled same as holotype; 7 specimens labeled: \ GUATEMALA: El Progreso: Cerro Pinalón, 15.08411-89.93239 ±57m, 2715m, 1-V-2009 ex. sifted leaf litter, cloud forest, LLAMA09 Wm-B-01-1-05 \ KUNHM \; 1 specimen labeled: \ GUAT.: EL PROGRESO: 19.6km.N.Estancia de la Virgen, 2000m, Finca la Illuciones, 24.VI.1993, R.Anderson, cloud for. litter, 93-13C \ CMNC \.

###### Type locality.

Guatemala, El Progreso Department, Cerro Pinalón.

###### Etymology.

The specific epithet is a Latinized eponym in the genitive case, and is based on the surname of John T. (Jack) Longino, Professor of the Biology Department of the University of Utah, and one of Co-PI’s of the LLAMA project, which provided the material on which the description of this species is based.

###### Recognition.

Adults of this new species are distinguished from those of other members of the *integripennis* species group by the large size, distinctive shape of the pronotum with very wide basal margin, and the proepisternum with evident microsculpture. Males and females of *Geocharidius longinoi* are distinguished from those of other members of the *integripennis* species group by the structure of the median lobe and the shape of spermatheca, respectively.

###### Description.

Size. Medium to large for genus (SBL range 1.34–1.51 mm, mean 1.41±0.071mm, n=12).

Habitus. Body form (Fig. 12C) moderately convex, elongate ovoid, general proportions (WE/SBL 0.38±0.008) and proportions of head (WH/WPm 0.71±0.012) average for group, pronotum markedly wide compared to elytra (WPm/WE 0.80±0.013).

Color. Body brunneorufous, appendages testaceous.

Microsculpture. Mesh pattern of irregularly isodiametric sculpticells present over all dorsal surfaces of head and elytra. Pronotum smooth (without evident microsculpture). Proepisternum with evident microsculpture.

Mouthparts. Maxillae and labium (Fig. 5E).

Prothorax. Pronotum slightly transverse (WPm/LP 1.25±0.019), with lateral margins slightly constricted posteriorly (WPm/WPp 1.29±0.018). Posterior angles slightly obtuse (100–110°). Width between posterior angles greater than between anterior angles (WPa/WPp 0.94±0.020).

Elytra. Moderately convex, slightly depressed along suture, moderately wide (WE/LE 0.66±0.020), without traces of striae. Humeri rounded, in outline forming right angle with longitudinal axis of body. Lateral margins convex, evenly divergent at basal forth, evenly rounded to apex in apical third.

Legs. Mesotibia (Fig. 7C). Metatibia (Fig. 7G).

Male genitalia. Median lobe (Fig. 13R) with shaft long, apically slightly widened, apex small and narrowly rounded. Ventral margin straight. Dorsal sclerites of internal sac (Fig. 13R) in form of long waved ribbon, tapered apically and slightly dilated basally, where sclerite forms small hook-like extension. Right paramere with short apical constriction (Fig. 13T). Left paramere with rather short and narrow apical constriction (Fig. 13S). Ring sclerite with triangularly rounded handle (Fig. 16F).

Female internal genitalia. Spermatheca sclerotized, elongate, subparallel, almost straight, with long cornu and short nodulus (Fig. 17F). Length of spermathecal gland less than length of spermatheca. Spermathecal duct not coiled.

###### Geographical distribution.

This species is known only from Cerro Pinalón, part of the Sierra de las Minas range of Guatemala (Fig. 22, green squares).

###### Way of life.

Specimens were extracted from cloud forest litter at elevations of 2000–2750 m.

###### Relationships.

The shape of handle of the ring sclerite (Fig. 16F) and the structure of dorsal sclerites of the internal sac (Fig. 13R) suggest a relationship with the Honduran *Geocharidius celaquensis* (Figs 20B and 19E), described below.

##### Geocharidius minimus sp. n.

Taxon classificationAnimaliaColeopteraCarabidae

http://zoobank.org/38CA85F3-81F4-4B3F-B23B-EB29892CEDAD

Figs 3E, G , 5F , 6D–E , 7B , 12H , 13G–J , 16C , 17C , 22 , 23

###### Type material.

HOLOTYPE, a male, in KUNHM, glued on cardboard, labeled: \ GUATEMALA: Sacatepéquez: 5km SE Antigua, 14.53577 -90.69428±200m, 2150m, 10-VI-2009, ex. sifted leaf litter, hardwood forest LLAMA09 Wa-B-08-1-all \ KUNHM \ HOLOTYPE *Geocharidius minimus* Sokolov and Kavanaugh 2014 [red label] \. PARATYPES: A total of 121 specimens (6 males and 4 females were dissected), deposited in CAS and KUNHM; 53 specimens labeled same as holotype; 11 specimens labeled: \ GUATEMALA: Sacatepéquez: 5km SE Antigua, 14.53439 -90.69340±36m, 2175m, 11-VI-2009, ex. sifted leaf litter, hardwood forest LLAMA09 Wm-B-08-2-08 \ KUNHM \; 1 specimen labeled: \ GUATEMALA: Sacatepéquez: 5km SE Antigua, 14.53666 -90.69491±255m, 2140m, 10-VI-2009, ex. sifted leaf litter, hardwood forest LLAMA09 Wm-B-08-1-07 \ SEMC0896573 KUNHM-ENT \; 48 specimens labeled: \ GUATEMALA: Sacatepéquez: 5km SE Antigua, 14.53482-90.69398±33m, 2175m, 10-VI-2009, ex. sifted leaf litter, hardwood forest LLAMA09 Wm-B-08-1-04 \ SEMC0888829 KUNHM-ENT \; 4 specimens labeled: \ GUATEMALA: Suchitepéquez: 4km S Vol. Atitlán, 14.55311- 91.19337 ±35m, 1750m, 15-VI-2009 ex sifted leaf litter, cloud forest, LLAMA09 Wm-B-09-2-02 1 \ KUNHM \; 2 specimens labeled: GUATEMALA: Suchitepéquez: 4km S Vol. Atitlán, 14.54915- 91.19055 ±200m, 1625m, 15-VI-2009 ex sifted leaf litter, cloud forest, LLAMA09 Wa-B-09-1-all \ SEMC0889856 KUNHM-ENT \; 2 specimens labeled: \ GUATEMALA: Suchitepéquez: 4km S Vol. Atitlán, 14.55972- 91.18951 ±27m, 2164m, 17-VI-2009 ex sifted leaf litter, cloud forest, LLAMA09 Wm-B-09-2-06 \ SEMC0896573 KUNHM-ENT \.

###### Type locality.

Guatemala, Sacatepéquez Department, 5 km SE of Antigua.

###### Etymology.

The specific epithet is a Latin adjective, *minimus* (superlative of *parvus*), in the masculine form, meaning “*smallest*”.

###### Recognition.

Adults of this new species are distinguished from those of other members of the *integripennis* species group by the combination of small size, elongate habitus and smooth proepisternum. Males and females of *Geocharidius minimus* are distinguished from those of other members of the *integripennis* species group by the structure of the median lobe and the shape of spermatheca, respectively.

###### Description.

Size. Small for genus (SBL range 1.11–1.24 mm, mean 1.18±0.041 mm, n=26).

Habitus. Body form (Fig. 12H) moderately convex, elongate, general proportions narrow (WE/SBL 0.37±0.009), proportions of head (WH/WPm 0.75±0.016) and pronotum (WPm/WE 0.80±0.015) wide for group.

Color. Body rufotestaceous, appendages testaceous.

Microsculpture. Mesh pattern of irregularly isodiametric sculpticells present over all dorsal surfaces of head and elytra. Pronotum and proepisternum smooth (without evident microscupture).

Mouthparts. Maxillae and labium (Fig. 5F).

Prothorax. Pronotum moderately narrow (WPm/LP 1.24±0.027), with lateral margins markedly constricted posteriorly (WPm/WPp 1.35±0.022). Posterior angles obtuse (110–120°). Width between anterior angles greater than between posterior angles (WPa/WPp 1.06±0.024).

Pterothorax (Fig. 3E).

Elytra. Moderately convex, slightly depressed along suture, moderately narrow (WE/LE 0.64±0.017), without traces of striae. Humeri rounded, in outline forming right angle with longitudinal axis of body. Lateral margins subparallel, evenly divergent at basal fifth, evenly rounded to apex in apical fourth.

Legs. Mesotibia (Fig, 7B). Protarsus (Figs 6D–E).

Abdomen. Ventrites 3-5 (Fig. 3G).

Male genitalia. Median lobe (Fig. 13G) with shaft moderately long, subparallel, apex small and narrowly rounded. Ventral margin almost straight. Dorsal sclerites of internal sac in form of a long fig, tapered apically as a long flagellum, and gradually widen towards narrow and rounded basal end extended through basal orifice and bent laterally (Fig. 13G–H). Right paramere with short apical consriction (Fig. 13J). Left paramere moderately long, with rather short apical constriction (Fig. 13I). Ring sclerite with handle triangular, slightly asymmetrical, pointed apically (Fig. 16C).

Female internal genitalia. Spermatheca sclerotized, fusiform, only slightly dilated apically, straight, with cornu and nodulus of approximately equal length (Fig. 17C). Lengths of spermathecal gland and spermatheca equal. Spermathecal duct not coiled.

###### Geographical distribution.

This species is known from the slopes of two volcanos, Agua and Atitlán, in Sacatepéquez and Suchitepéquez Departments of Guatemala, respectively (Fig. 22, green flowers).

###### Way of life.

Specimens were collected by sifting litter in hardwood and cloud forests at middle and high elevations of 1600 and 2200 m, respectively.

###### Relationships.

The shape of dorsal sclerites of the internal sac (Fig. 13G) of males suggests a distant relationship with *Geocharidius erwini* (Fig. 13D), described above, whereas the shape of the handle of the ring sclerite (Fig. 16C) of males suggests relationships with the Guatemalan *Geocharidius antigua* (Fig. 20A), described above, and the Honduran *Geocharidius disjunctus* (Fig. 20E), described below.

#### Species from Honduras

##### Geocharidius celaquensis sp. n.

Taxon classificationAnimaliaColeopteraCarabidae

http://zoobank.org/2AE3AACE-E66B-4F68-B8D8-AF5974E29BC6

**Figures 18.**
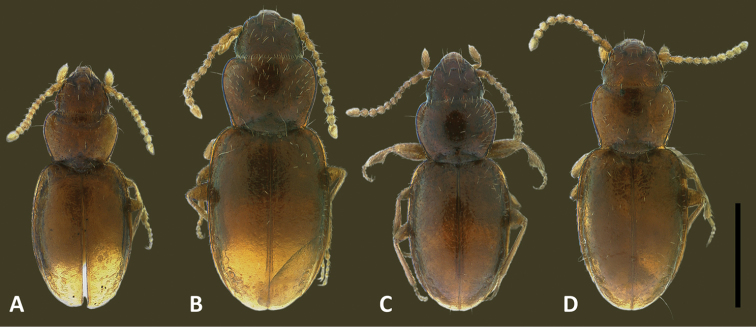
Digital images of habitus of Honduran *Geocharidius* species. **A** *Geocharidius celaquensis* (HONDURAS, Lempira, Celaque National Park), paratype **B** *Geocharidius lencanus* (HONDURAS, Lempira, Celaque National Park), paratype **C** *Geocharidius comayaguanus* (HONDURAS, Comayagua, Comayagua), paratype **D** *Geocharidius disjunctus* (HONDURAS, Francisco Morazán, La Tigra National Park), holotype. Scale = 0.5mm.

**Figures 19.**
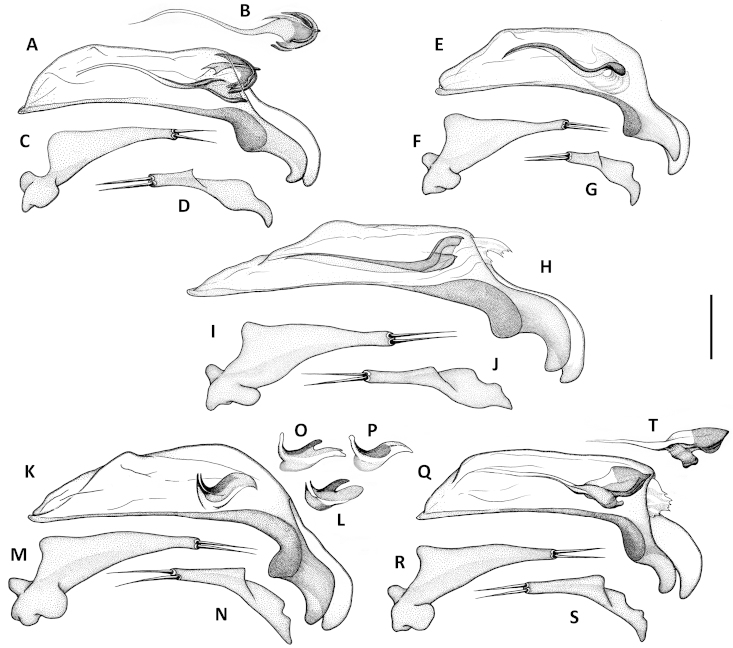
Line drawings of aedeagus of Guatemalan and Honduran *Geocharidius* species. **A–D** *Geocharidius antigua* (GUATEMALA, Sacatepéquez, Antigua), holotype: **A** median lobe with internal sac and dorsal sclerites, right lateral aspect **B** dorsal sclerite of median lobe, dorsal aspect **C** left paramere, left lateral aspect **D** right paramere, right lateral aspect **E–G** *Geocharidius celaquensis* (HONDURAS, Lempira, Celaque National Park), holotype: **E** median lobe with internal sac and dorsal sclerites, right lateral aspect **F** left paramere, left lateral aspect **G** right paramere, right lateral aspect **H–J** *Geocharidius lencanus* (HONDURAS, Lempira, Celaque National Park), paratype: **H** median lobe with internal sac and dorsal sclerites, right lateral aspect **I** left paramere, left lateral aspect **J** right paramere, right lateral aspect **K–N** *Geocharidius comayaguanus* (HONDURAS, Comayagua, Comayagua), paratype: **K** median lobe with internal sac and dorsal sclerites, right lateral aspect **L** variation in a shape of dorsal sclerite of internal sac, right lateral aspect **M** left paramere, left lateral aspect **N** right paramere, right lateral aspect **O–P** *Geocharidius comayaguanus* (HONDURAS, La Paz, Guajicuiro): variations in a shape of dorsal sclerite of internal sac, right lateral aspect **Q–S** *Geocharidius disjunctus* (HONDURAS, Francisco Morazán, La Tigra National Park), holotype: **Q** median lobe with internal sac and dorsal sclerites, right lateral aspect **R** left paramere, left lateral aspect **S** right paramere, right lateral aspect. **T** *Geocharidius disjunctus* (HONDURAS, Yoro, Pico Pijol National Park): shape of dorsal sclerite of median lobe, right lateral aspect. Scale = 0.05mm.

**Figures 20.**
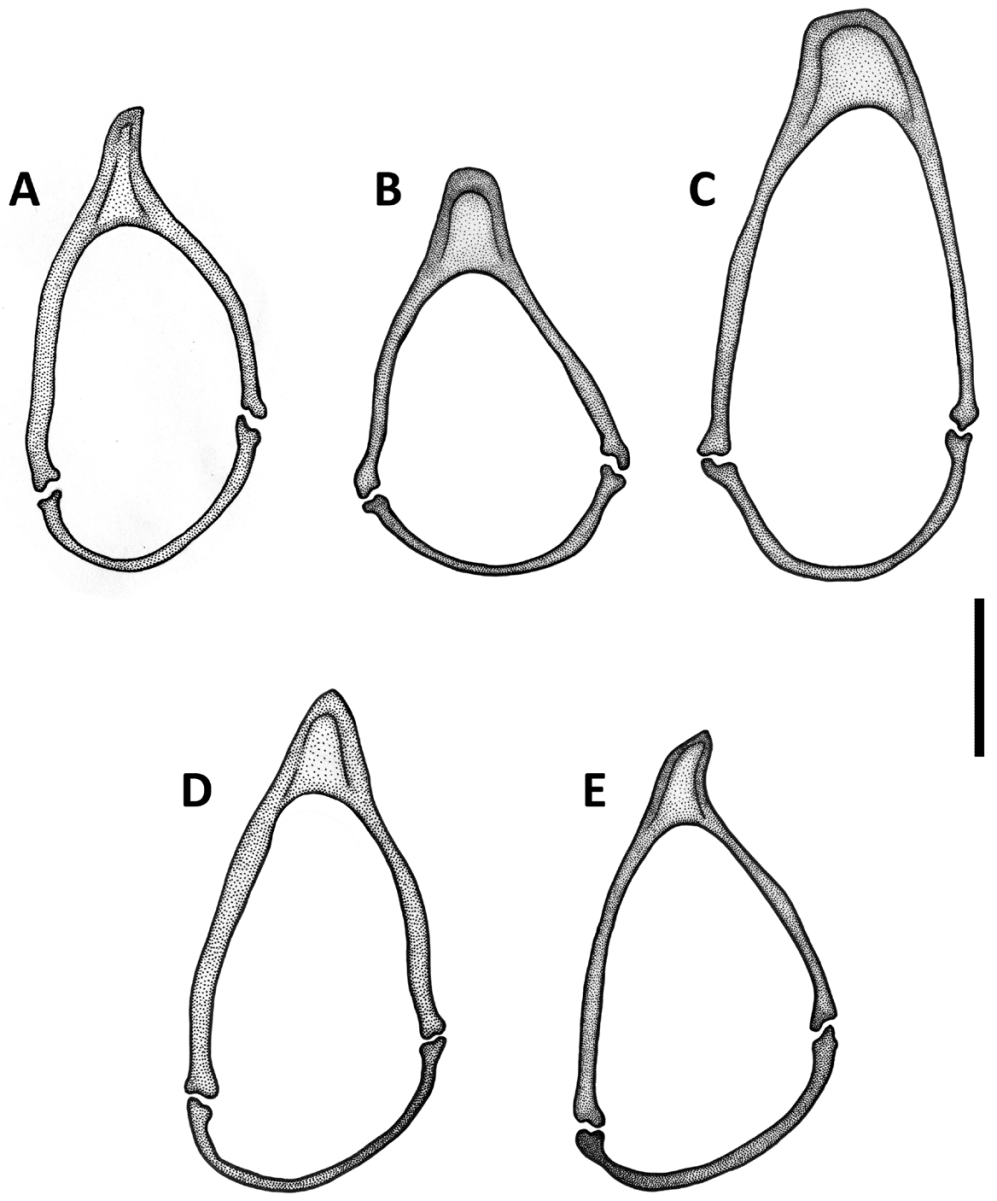
Line drawings of ring sclerite of Guatemalan and Honduran *Geocharidius* species, male genitalia, dorsal aspect. **A** *Geocharidius antigua* (GUATEMALA, Sacatepéquez, Antigua), holotype **B** *Geocharidius celaquensis* (HONDURAS, Lempira, Celaque National Park), holotype **C** *Geocharidius lencanus* (HONDURAS, Lempira, Celaque National Park), paratype **D** *Geocharidius comayaguanus* (HONDURAS, Comayagua, Comayagua), paratype **E** *Geocharidius disjunctus* (HONDURAS, Francisco Morazán, La Tigra National Park), holotype. Scale = 0.1mm.

**Figures 21.**
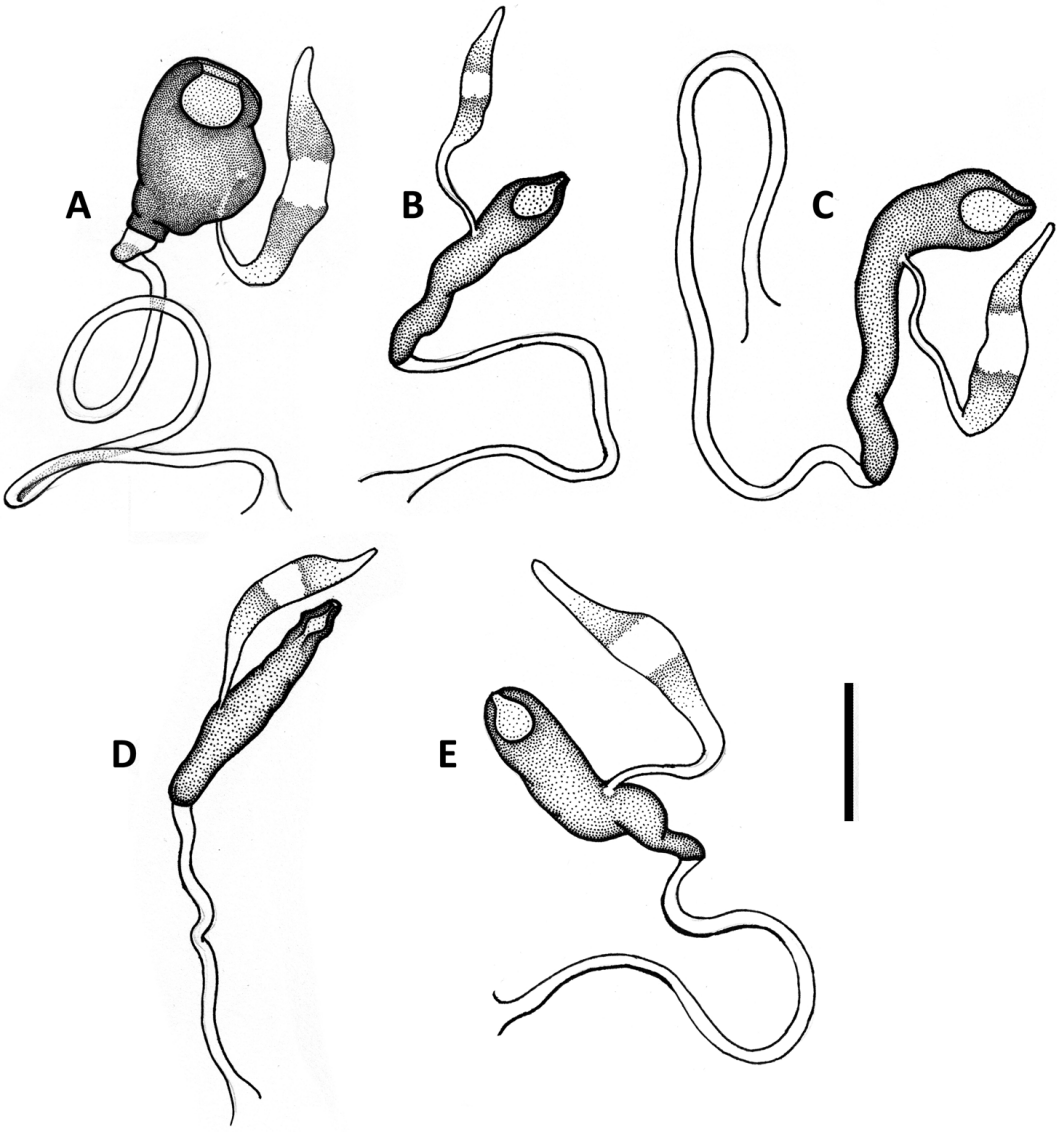
Line drawings of spermatheca of Guatemalan and Honduran *Geocharidius* species. **A** *Geocharidius antigua* (GUATEMALA, Sacatepéquez, Antigua), paratype **B** *Geocharidius celaquensis* (HONDURAS, Lempira, Celaque National Park), paratype **C** *Geocharidius lencanus* (HONDURAS, Lempira, Celaque National Park), paratype **D** *Geocharidius comayaguanus* (HONDURAS, Comayagua, Comayagua), paratype **E** *Geocharidius disjunctus* (HONDURAS, Yoro, Pico Pijol National Park). Scale = 0.05mm.

**Figure 22.**
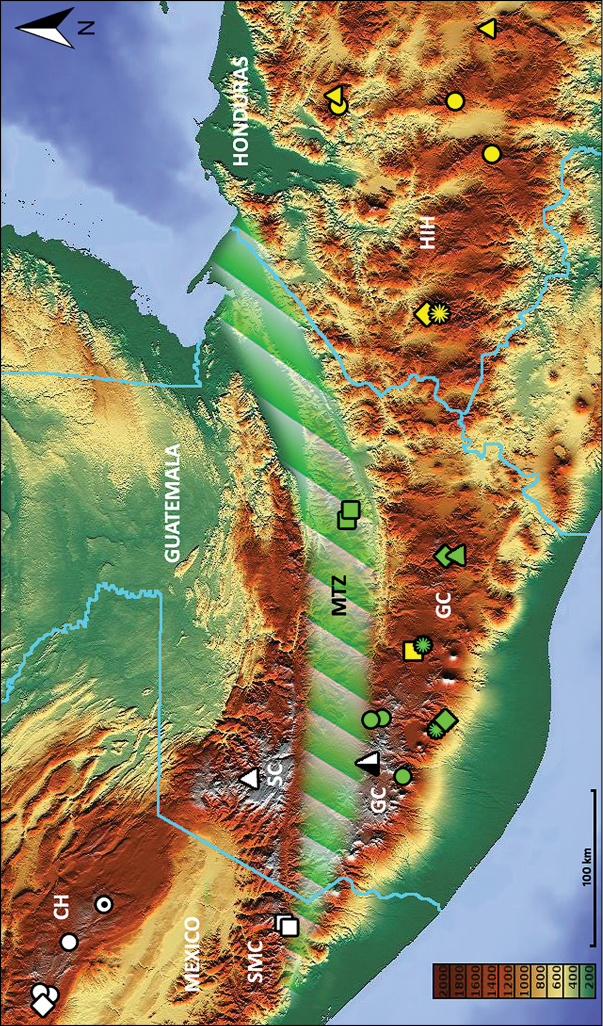
Map of southern Mexico, Guatemala and adjacent part of Honduras, showing positions of locality records for the species of *Geocharidius*: white diamond, *Geocharidius andersoni*; white circles (black point in a circle shows “terra typica” for the species), *Geocharidius zullinii*; white squares, *Geocharidius vignatagliantii*; white triangle, *Geocharidius gimlii*; black and white triangle, *Geocharidius integripennis*; green squares, *Geocharidius longinoi*; green circles, *Geocharidius erwini*; green flowers, *Geocharidius minimus*; green diamonds, *Geocharidius balini*; green triangle, *Geocharidius jalapensis*; yellow quadrangle, *Geocharidius antigua*; yellow diamond, *Geocharidius lencanus*; yellow flower, *Geocharidius celaquensis*; yellow triangles, *Geocharidius disjunctus*; yellow circles, *Geocharidius comayaguanus*. Physiographic features: CH, Chiapas Highlands; GC, Guatemalan Cordillera; HIH, Honduran Interior Highlands; MTZ, Motagua Fault Zone; SC, Sierra de los Cuchumatanes; SMC, Sierra Madre de Chiapas. Elevation scale bar is given in meters.

**Figures 23.**
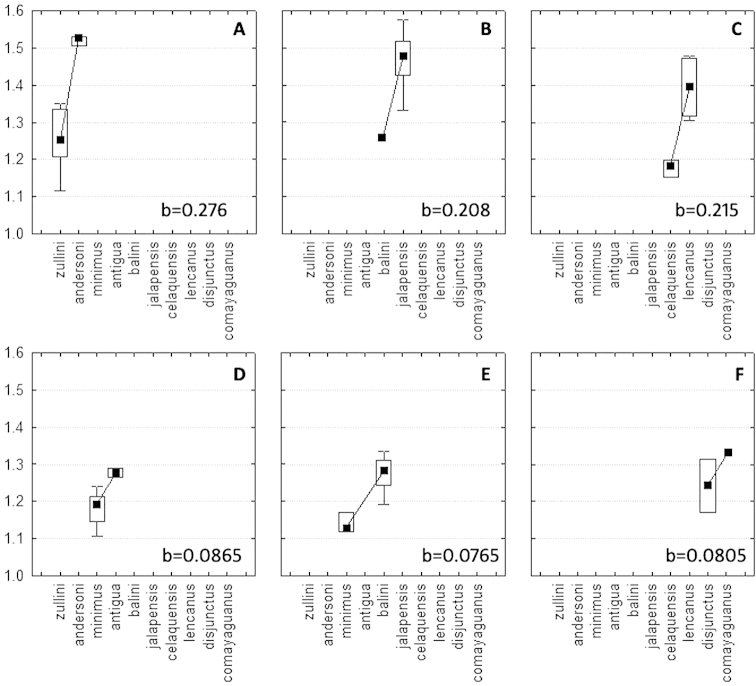
Diagrams illustrating size variation for sympatric pairs of closely and remotely related species of the *integripennis* group at different geographical localities of Nuclear Central America. Closely related pairs (for shared characters see subchapters on Relationships in the text for corresponding species): **A** Chiapas Highlands, Mexico **B** Mataquescuintla, Guatemala **C** Celaque National Park, Honduras. Remotely related species: **D** Volcano Agua, Guatemala **E** Volcano Atitlan, Guatemala **F** Pico Pijol National Park, Honduras. Legend: black dot – median; box – 25–75% range of values; whiskers – non-outlier range of values; b – coefficient of regression.

Figs 18A , 19E–G , 20B , 21B , 22 , 23

###### Type material.

HOLOTYPE, a male, in CMNC, point-mounted, dissected, labeled: \ HONDURAS: Lempira Dept., P.N. Celaque, nr. Gracias, Campamiento Naranjo, 2500m, 14°32.7'N, 88°39.7'W, 12–13.V.2002, cloud forest litter R. Anderson, 2002-020C \ CMNC \ HOLOTYPE *Geocharidius celaquensis* Sokolov and Kavanaugh 2014 [red label] \. PARATYPES: A total of 2 females (both were dissected), deposited in CAS and KUNHM; labeled same as holotype, except label of the holder: SEMC0… KUNHM-ENT \.

###### Type locality.

Honduras, Lempira Department, Celaque National Park.

###### Etymology.

The specific epithet is a Latinized adjective in the masculine form based on the name of Celaque National Park, from which the new species is described.

###### Recognition.

Adults of this new species are distinguished from those of other members of the *integripennis* species group by their small size, fully microsculptured dorsal body surface and pronotum with wide basal margin. Males and females of *Geocharidius celaquensis* are distinguished from those of the other members of the *integripennis* species group by the structure of the median lobe and the shape of spermatheca, respectively.

###### Description.

Size. Small for genus (SBL range 1.15–1.20 mm, mean 1.18±0.023mm, n=3).

Habitus. Body form (Fig. 18A) moderately convex, ovoid, general proportions (WE/SBL 0.40±0.005), proportions of head (WH/WPm 0.73±0.016) and pronotum (WPm/WE 0.78±0.015) moderately wide.

Color. Body rufotestaceous, appendages testaceous.

Microsculpture. Mesh pattern of irregularly isodiametric sculpticells present over all dorsal surfaces of head, pronotum and elytra. Proepisternum also with evident microsculpture.

Prothorax. Pronotum markedly transverse (WPm/LP 1.32±0.025), with lateral margins markedly constricted posteriorly (WPm/WPp 1.35±0.002). Posterior angles obtuse (110–120°). Width between posterior angles slightly greater than between anterior angles (WPa/WPp 1.04±0.004).

Elytra. Moderately convex, slightly depressed along suture, moderately wide (WE/LE 0.68±0.015), without traces of striae. Humeri rounded, in outline forming right angle with longitudinal axis of body. Lateral margins convex, evenly divergent at basal third, evenly rounded to apex in apical third.

Male genitalia. Median lobe (Fig. 19E) with shaft subparallel, apex small and narrowly rounded. Ventral margin almost straight. Dorsal sclerites of internal sac in form of long, waved ribbon, tapered apically and slightly dilated and narrowly rounded basally. Right paramere with short apical constriction (Fig. 19G). Left paramere with long and narrow apical constriction (Fig. 19F). Ring sclerite with handle triangular, widely rounded at apex (Fig. 20B).

Female internal genitalia. Spermatheca sclerotized, fusiform, slightly dilated apically, straight, with short cornu and long nodulus (Fig. 21B). Length of spermathecal gland less than length of spermatheca. Spermathecal duct not coiled.

###### Geographical distribution.

This species is known only from Celaque National Park, part of the Cerro las Minas range of Honduras (Fig. 22, yellow flower).

###### Way of life.

Specimens were extracted from cloud forest litter at an elevation of 2500 m.

###### Relationships.

The shape of dorsal sclerites of the internal sac (Fig. 19E) in males and the point of the attachment of the spermathecal gland (Fig. 21B) in females suggest that this species is closely related to *Geocharidius lencanus* (Figs 19H and 21C), described below, and perhaps also, but more remotely, to the Guatemalan *Geocharidius longinoi* (Figs 13R and 17F), described above.

##### Geocharidius comayaguanus sp. n.

Taxon classificationAnimaliaColeopteraCarabidae

http://zoobank.org/CC2857E4-7428-4FC0-A2BC-CFA5A571E8C3

Figs 2C , 3C , 3F , 6H , 7D, H , 18C , 19K–P , 20D , 21D , 22 , 23

###### Type material.

HOLOTYPE, a male, in CMNC, point-mounted, dissected, labeled: \ HONDURAS: Comayagua, 18km ENE Comayagua, 1950m, 20.VIII.1994, S. & J. Peck, wet oak-pine forest litter, S&JPeck 1994-52 \ CMNC \ HOLOTYPE *Geocharidius comayaguanus* Sokolov and Kavanaugh 2014 [red label] \. PARATYPES: A total of 23 specimens (6 males and 2 females were dissected), deposited in CAS, CMNC and KUNHM; 4 specimens labeled same as holotype; 5 specimens labeled: \ HONDURAS: Comayagua, Comayagua (18km E.N.E.), 1950m, 20.VIII.1994, S. Peck wet oak-pine forest litter, SBP 94-52 \ CMNC \; 8 specimens labeled: HONDURAS: La Paz Dept. Tutule, Res. Biol. Guajiquiro, 14°10'N, 87°50'W, 2130m, 7-V-2002, R.Anderson, cloud forest litter, RSA2002-010 \ SM0… KUNHM-ENT \; 1 specimen labeled: HONDURAS: LA PAZ: Tutule, Res. Biol. Guajiquiro, 14°10'N, 87°50'W, 2130m, 7.V.2002, R.Anderson, cloud forest litter, 2002-010H \ CMNC \; 1 specimen labeled: \ HONDURAS: LA PAZ: Tutule, Res. Biol. Guajiquiro, 14°10'N, 87°50'W, 2130m, 7.V.2002, R.Anderson, cloud forest litter, 2002-010D \ CMNC \; 1 specimen labeled: \ HONDURAS: LA PAZ: Tutule, Res. Biol. Guajiquiro, 14°10'N, 87°50'W, 2130m, 7.V.2002, R.Anderson, cloud forest litter, 2002-010E \ CMNC \; 2 specimens labeled: \ HONDURAS: LA PAZ: Tutule, Res. Biol. Guajiquiro, 14°10', N87°50'W, 2130m, 7.V.2002, R.Anderson, cloud forest litter, 2002-010I \ CMNC \; 1 specimen labeled: \ HONDURAS: Yoro Dept., P.N. Pico Pijol, 1300m, 15°09.4', N87°37.6'W, 11.V.2002, R. Anderson, upper montane forest litter, 2002-017A\ CMNC.

###### Type locality.

Honduras, Comayagua Department, 18 km ENE of Comayagua.

###### Etymology.

The specific epithet is a Latinized adjective in the masculine form based on the name of the city of Comayagua, from the vicinity of which the new species is described.

###### Recognition.

Adults of this species are practically indistinguishable externally from those of *Geocharidius disjunctus*, described below, and are distinguished from the latter, as from those of the other members of the *integripennis* species group, by the structure of the male median lobe and the shape of spermatheca in females.

###### Description.

Size. Small to medium for genus (SBL range 1.19–1.34 mm, mean 1.28±0.072mm, n=20).

Habitus. Body form (Fig. 18C) moderately convex, ovoid, general proportions (WE/SBL 0.40±0.011), proportions of head (WH/WPm 0.74±0.017) and pronotum (WPm/WE 0.78±0.018) moderately wide.

Color. Body brunneorufous, appendages testaceous.

Microsculpture. Mesh pattern of irregularly isodiametric sculpticells present over all dorsal surfaces of head and elytra. Pronotum and proepisternum smooth (without evident microsculpture).

Prothorax. Pronotum moderately transverse (WPm/LP 1.29±0.024), with lateral margins markedly constricted posteriorly (WPm/WPp 1.35±0.027). Posterior angles obtuse (110–120°). Width between posterior angles equal to the width between anterior angles (WPa/WPp 1.02±0.026). Ventral aspect (Fig. 3C).

Pterothorax (Fig. 3F).

Elytra (Fig. 2C). Moderately convex, slightly depressed along suture, moderately wide (WE/LE 0.68±0.022), without traces of striae. Humeri rounded, in outline forming right angle with longitudinal axis of body. Lateral margins convex, evenly divergent at basal third, evenly rounded to apex in apical third.

Legs. Protibia (Fig. 6H). Mesotibia (Fig. 7D). Metatibia (Fig. 7H).

Male genitalia. Median lobe (Fig. 19K) with shaft long and dorsally convex, apex small and narrowly rounded. Ventral margin straight. Dorsal sclerites of internal sac small, in form of a short hook-like fig, slightly varied among different populations (Fig. 19K–L, O–P). Right paramere with long apical constriction (Fig. 19N). Left paramere with long and narrow apical constriction (Fig. 19M). Ring sclerite with handle triangular, pointed at apex (Fig. 20D).

Female internal genitalia. Spermatheca sclerotized, fusiform, slightly tapered apically, straight, with cornu and nodulus of equal length (Fig. 21D). Length of spermathecal gland less than length of spermatheca. Spermathecal duct not coiled.

###### Geographical distribution.

This species is known from La Paz, Comayagua and Yoro Departments, thus having a range that crosses nearly the entire Honduran Interior Highlands from the Pacific to the Antlantic slope (Fig. 22, yellow circles).

###### Way of life.

Specimens were collected in litter samples from cloud, upper montane and wet oak-pine forests at middle and high elevations of 1300 and 2130 m, respectively.

###### Relationships.

This species is unique within the *integripennis* species group in the shape of dorsal sclerites of the internal sac (Fig. 19K) of males and of the spermatheca (Fig. 21D) of females. Hence, *Geocharidius comayaguanus* appears to be only remotely related to the other members of the species group.

##### Geocharidius disjunctus sp. n.

Taxon classificationAnimaliaColeopteraCarabidae

http://zoobank.org/A0E6C548-3EC5-40B6-933F-414125D61068

Figs 18D , 19Q–T , 20E , 21E , 22 , 23

###### Type material.

HOLOTYPE, a male, in CMNC, point-mounted, dissected, labeled: \ HONDURAS: FRANC. MOR: P.N. La Tigra, 23.2km N Tegucigalpa, 15.VIII.1994-201A, 2100m, R.Anderson, cloud forest litter berlese \ CMNC \ HOLOTYPE *Geocharidius disjunctus* Sokolov and Kavanaugh 2014 [red label] \. PARATYPES: A total of 2 specimens (both were dissected), deposited in CAS and CMNC; 1 male labeled: \ HONDURAS: Yoro Dept., P.N. Pico Pijol, 1400m, 15°09.4'N87°37.6'W, 11.V.2002, R. Anderson, upper montane forest litter, 2002-016C\ CMNC \; 1 female labeled: \ HONDURAS: Yoro Dept., P.N. Pico Pijol, 1300m, 15°09.4'N87°37.6'W, 11.V.2002, R. Anderson, upper montane forest litter, 2002-017A \ CMNC \.

###### Type locality.

Honduras, Francisco Morazán, La Tigra National Park.

###### Etymology.

The specific epithet is a Latin adjective, *disjunctus*, in the masculine form, meaning “*separated*”, and refers to the species distinctness from the sympatric *Geocharidius comayaguanus*, described above.

###### Recognition.

Adults of this new species are practically indistinguishable from those of the sympatric *Geocharidius comayaguanus* in body shape. Males and females of *Geocharidius disjunctus* are distinguished from those of the other members of the *integripennis* species group by the structure of the median lobe and the shape of spermatheca, respectively.

###### Description.

Size. Small to medium for genus (SBL range 1.17–1.36 mm, mean 1.28±0.101 mm, n=3).

Habitus. Body form (Fig. 18D) moderately convex, elongate ovoid, general proportions (WE/SBL 0.38±0.005) and proportions of head (WH/WPm 0.74±0.020) and pronotum (WPm/WE 0.78±0.018) average for group.

Color. Body brunneorufous, appendages testaceous.

Microsculpture. Mesh pattern of irregularly isodiametric sculpticells present over all dorsal surfaces of head and elytra. Pronotum and proepisternum smooth (without evident microsculpture).

Prothorax. Pronotum moderately transverse (WPm/LP 1.28±0.010), with lateral margins moderately constricted posteriorly (WPm/WPp 1.33±0.004). Posterior angles slightly obtuse (100–110°). Width between posterior angles equal to width between anterior angles (WPa/WPp 1.00±0.022).

Elytra. Moderately convex, slightly depressed along suture, moderately wide (WE/LE 0.65±0.015), without traces of striae. Humeri broadly rounded, in outline forming right angle with longitudinal axis of body. Lateral margins convex, evenly divergent at basal third, evenly rounded to apex in apical third.

Male genitalia. Median lobe of aedeagus (Fig. 19Q) with shaft long and subparallel, apex small and rounded. Ventral margin straight. Dorsal sclerites of internal sac in form of a long fig, tapered apically into a long flagellum, and abruptly widened basally, with a ventral appendix and pointed semicircular enlargement near basal orifice (Fig. 19Q–T). Right paramere with long and narrow apical constriction (Fig. 19S). Left paramere with long and narrow apical constriction (Fig. 19R). Ring sclerite with handle triangular, slightly asymmetrical and pointed apically (Fig. 20E).

Female internal genitalia. Spermatheca sclerotized, fusiform, almost straight, tapered basally, with cornu and nodulus of approximately equal length (Fig. 21E). Length of spermathecal gland greater than length of spermatheca. Spermathecal duct coiled.

###### Geographical distribution.

This species is known only from two remote localities in the Honduran Interior Highlands, situated in Yoro and Francisco Morazán Departments.(Fig. 22, yellow triangles).

###### Way of life.

Specimens were collected by sifting cloud and upper montane forest litter at middle to high elevations (1300–2100 m).

###### Relationships.

The shape of dorsal sclerites of the internal sac (Fig. 19Q) of males suggests that this species is closely related to the Guatemalan *Geocharidius antigua* (Fig. 19A), described above

##### Geocharidius lencanus sp. n.

Taxon classificationAnimaliaColeopteraCarabidae

http://zoobank.org/B41C1418-E0FB-4896-BE0F-827FE08A84D1

Figs 5C , 6G , 18B , 19H–J , 20C , 21C , 22 , 23

###### Type material.

HOLOTYPE, a male, in KUNHM, point-mounted, dissected, labeled: \ HONDURAS: Lempira Dept., P.N. Celaque, nr. Gracias, Campamiento Naranjo, 14°32.7'N, 88°39.7'W, 2500m, 12-13-V-2002, cloud forest litter R. Anderson, RSA2002-020 \ SM0… KUNHM-ENT \ HOLOTYPE *Geocharidius lencanus* Sokolov and Kavanaugh 2014 [red label] \. PARATYPES: A total of 6 specimens (3 males and 2 females were dissected), deposited in CAS, CMNC and KUNHM; 4 specimens labeled same as holotype; 2 specimens labeled: \ HONDURAS: Lempira Dept., P.N. Celaque, nr. Gracias, Campamiento Naranjo, 2500m, 14°32.7', N88°39.7'W, 12–13.V.2002, cloud forest litter R. Anderson, 2002-020E \ CMNC \.

###### Type locality.

Honduras, Lempira Department, Celaque National Park.

###### Etymology.

The specific epithet is a Latinized adjective in the masculine form based on the name of the indigenous people, the *Lenca*, living in the territory of Celaque National Park during historic times.

###### Recognition.

Externally, members of this species represent a larger version of *Geocharidius celaquensis* adults, described above. Adults of *Geocharidius lencanus* are distinguished from those of other members of the *integripennis* species group by the following combination of external characters: medium to large size, rather wide habitus and fully microsculptured dorsal body surface. Males and females of *Geocharidius lencanus* are distinguished from those of the other members of the *integripennis* species group by the structure of the median lobe and the shape of spermatheca, respectively.

###### Description.

Size. Medium to large for genus (SBL range 1.30–1.47 mm, mean 1.39±0.091mm, n=4).

Habitus. Body form (Fig. 18B) moderately convex, ovoid, general proportions (WE/SBL 0.40±0.006), proportions of head (WH/WPm 0.73±0.008) and pronotum (WPm/WE 0.77±0.010) average for group.

Color. Body rufotestaceous, appendages testaceous.

Microsculpture. Mesh pattern of irregularly isodiametric sculpticells present over all dorsal surfaces of head, pronotum and elytra. Proepisternum also with evident microsculpture.

Mouthparts. Maxillae and labium (Fig. 5C).

Prothorax. Pronotum moderately transverse (WPm/LP 1.28±0.009), with lateral margins moderately constricted posteriorly (WPm/WPp 1.34±0.019). Posterior angles slightly obtuse (100–110°). Width between posterior angles nearly equal to the width between anterior angles (WPa/WPp 1.01±0.012).

Legs. Protibia (Fig. 6G).

Elytra. Moderately convex, slightly depressed along suture, markedly wide (WE/LE 0.69±0.013), without traces of striae. Humeri rounded, in outline forming right angle with longitudinal axis of body. Lateral margins convex, evenly divergent in basal fourth, evenly rounded to apex in apical third.

Male genitalia. Median lobe (Fig. 19H) with shaft subparallel with a long attenuated preapical part, apex small and narrowly rounded. Ventral margin almost straight. Dorsal sclerites of internal sac in form of a long narrow fig, flagellum-like in apical two-thirds, and slightly dilated and curved dorsally in basal third. Right paramere with long and narrow apical constriction (Fig. 19J). Left paramere with long and narrow apical constriction (Fig. 19I). Ring sclerite with handle rectangularly rounded (Fig. 20C).

Female internal genitalia. Spermatheca sclerotized, fusiform, slightly dilated and rectangularly bent apically, with short cornu and long nodulus (Fig. 21C). Length of spermathecal gland less than length of spermatheca. Spermathecal duct not coiled.

###### Geographical distribution.

This species is known only from Celaque National Park, in the Cerro las Minas range of Honduras (Fig. 22, yellow diamond).

###### Way of life.

Specimens were collected by sifting cloud forest litter at an elevation of 2500 m.

###### Relationships.

The rectangular shape of the handle of the ring sclerite (Fig. 20C) in males and the shape of spermatheca (Fig. 21C) in females suggest a close relationship with *Geocharidius celaquensis* (Figs 20B, 21B), described above.

## Discussion

A comprehensive phylogenetic and biogeographic analysis of *Geocharidius* is postponed until a thorough revision of all species of the genus has been completed. Below we discuss only a few biogeographical and evolutionary issues, raised during our morphological and distributional studies of the *integripennis* group species.

### Biogeographical notes

Distributional records of the *integripennis* species group to date are represented in Table 1 and can be summarized as follows: The group includes mostly high elevation species: all 15 species live at elevations greater than 2000m, and only four of these also inhabit the 1300-2000m mid-elevation range. Physiographically (Fig. 22), species of the group inhabit the interior mountain ranges of the Chiapas (CH), Guatemalan (SC+MTZ) and Honduran Highlands (HIH) along with the coastal Sierra Madre de Chiapas (SMC) and its continuation as the Guatemalan Cordillera (GC). Geologically, these territories are part of the Maya Block of the North American and the Chortis Block of the Carribean Tectonic Plates, divided by the Motagua (or Motagua-Polochic) Fault Zone (Marschall 2007). The Motagua Fault Zone (Fig. 22, MTZ) has been identified as the most important physiographic barrier in Nuclear Central America, corresponding to many phylogeographic breaks in the distributions of different vertebrate taxa (Perdices et al. 2005, Conchero Pérez et al. 2006; Castoe et al. 2009, Daza et al. 2010, Hardy et al. 2013). For *Geocharidius* species, this zone separates montane areas with higher species diversity (to the south and east) from those with lower diversity (to the north and west).

Each of the six montane areas has its own unique assemblage of *Geocharidius* species, ranging in number from one to six species; and none of these species are shared between montane areas. Consequently, any faunal connections between them are through sister species rather than through conspecific populations; and this pattern has implications for the timing of past dispersal and vicariance events (i.e., suggesting somewhat greater antiquity for such events). As mentioned above, the Motagua Fault Zone is a major physiographic barrier limiting the present distributions of *integripennis* group species. A second evidently strong barrier is one between the faunas of the Guatemalan Cordillera and the Honduran Interior Highlands, separating the ranges of six and four species endemic to each of these regions, respectively. The headwater valleys of the Rio Paz to the south and Rio Motagua/Rio Shutague to the north, respectively, are linked by gaps in the intervening uplands that do not exceed 900m in elevation, creating a continuous break across these highlands that is 400m lower than the lowest elevations at which any *intergripennis* species in the region has been recorded.

Among *integripennis* group members, six species have quite wide ranges within their own montane area, while the other nine species are known from only one locality or from two very close localities (*Geocharidius longinoi*) within their area. Within-group diversity varies markedly between different parts of the region. Four of the six montane areas are inhabited by one or two species, the Honduran Interior Highlands by four species, and the Guatemalan Cordillera volcanic chain (Fig. 22, GC) by six species. Within these areas, such diversity is not based solely on a high number of locally restricted forms. For example, three of the six species of the Guatemalan Cordillera have rather wide ranges for *Geocharidius* specie*s.* This distributional pattern results in three cases of sympatry among the species within the Cordillera. MacVean and Schuster (1981) recorded similarly wide ranges for passalid beetle species and sympatry among them on volcanoes of the Guatemalan Cordillera.

Within the range of the *integripennis* species group, the Guatemalan Cordillera occupies a special place and can be characterized by the highest number of the species in total, the highest number of species with wide ranges and the highest number of localities in which sympatry has been recorded. This combination of parameters may indicate that, historically, this region played an important role as a staging area for immigrants and as an intersection of dispersal routes of *integripennis* group species dispersing between different areas.

Two cases of evident similarities in morphology of male and female genitalia between species inhabiting the Cordillera and their relatives outside the region seem to support the above mentioned assertion. The similarity in median lobe structure between the eastern Guatemalan *Geocharidius antigua* (Fig. 19A) and the Honduran *G disjunctus* (Fig. 19Q) is unequivocal; and presumably homologous structures in the median lobe of males of *Geocharidius integripennis* and *Geocharidius gimlii* (Fig. 15) are virtually identical. This leads us to consider these pairs as sister species. These examples connect the Cordilleran fauna (Fig. 22, GC) with faunas of the Honduran Highlands (HIH) and Sierra de los Cuchumatanes (SC), respectively, thus, supporting our evaluation of the role of the Guatemalan Cordillera as important in the dispersal history of the *integripennis* group.

Further, certain morphological similarities can be found between the west Guatemalan pair of species, *Geocharidius gimlii*, and *Geocharidius integripennis*, and among the Mexican trio of species, *Geocharidius andersoni*, *Geocharidius zullinii* and*Geocharidius vignatagliantii*. Males of all three Mexican species share a similar shape of the dorsal sclerites of the median lobe (Fig. 9), and a triangular handle of the ring sclerite (Fig. 10). At the same time, females of *Geocharidius vignatagliantii* can be connected with those of the west Guatemalan *Geocharidius integripennis* by the short, sclerotized, and basally swollen basally spermatheca (Figs 11B, 17A), while the dorsal sclerites of the median lobe of *Geocharidius andersoni* males (Fig. 9H) are somewhat similar to the shortened variant of the dorsal sclerites of *Geocharidius gimlii* males (Fig. 13A).

These examples suggest that the Sierra de los Cuchumatanes (Fig. 22, SC) may have served as an important dispersal route from the Pacific coastal Guatemalan Cordillera northward to the Chiapas Highlands (CH). Given its proximity to the Guatemalan Cordillera, the Sierra Madre de Chiapas (Fig. 22, SMC) would appear to have been a more likely dispersal route northward, but we have no evidence that this route has been used. It is worth noting that, based on morphology, all species of the group living to the north of the Motagua Fault Zone appear to be rather closely related to each other, whereas the species living to the south and to the east of the Motagua Fault Zone appear to represent several morphologically different lineages. Perhaps the Mexican representatives of the group are descendants of a comparatively recent dispersal event involving one of the southern lineages.

Searching for concordant taxon-area relationships in other taxa reveals other carabids with similar distribution patterns. The distribution pattern for species of the pterostichine subgenus *Percolaus* Bates, as described by Ball and Roughley (1982), is identical to the distribution pattern of the Mexican-west Guatemalan set of *Geocharidius*’ species and encompasses the Chiapas Highlands, the Sierra Madre de Chiapas, Cerro Maria Tecún and Sierra de los Cuchumatanes. Interestingly, these authors suggested that *Pterostichus (Percolaus) championi* Bates, from the Cerro Maria Tecún has its closest known relative in the Sierra de los Cuchumatanes, the same relationship we see between *Geocharidius integripennis*, presumably collected in the Cerro Maria Tecún, and *Geocharidius gimlii* from the Sierra de los Cuchumatanes. Also, the distribution patterns of three Mexican and one western Guatemalan *Geocharidius* (namely *Geocharidius andersoni*, *Geocharidius zullinii*, *Geocharidius vignatagliantii* and *Geocharidius gimlii* )correspond perfectly to the distribution pattern of the species of *Platynus jaegeri* group (namely *Platynus dilatipes* Liebherr, *Platynus robustus* (Chaudoir) and *Platynus strictinotum* Liebherr (Liebherr 1988).

**Table 1.** Montane areas in Nuclear Central America occupied by the species of the *Geocharidius integripennis* species group.

Species	Montane areas						Elevation range (in meters)	Number of localities
	CH	SMC	SC	MTZ	GC	HIH		
*Geocharidius andersoni*	X						2750	1
*Geocharidius zullinii*	X						2350–2600	6
*Geocharidius vignataglianti*		X					2050	1
*Geocharidius gimlii*			X				2780	1
*Geocharidius longinoi*				X			2000–2750	2
*Geocharidius integripennis*					X		3200	1
*Geocharidius balini*					X		1625–2400	2
*Geocharidius jalapensis*					X		2325–2660	1
*Geocharidius erwini*					X		2140–2760	4
*Geocharidius minimus*					X		1625–2175	2
*Geocharidius antigua*					X		2350	1
*Geocharidius disjunctus*						X	1300–2100	2
*Geocharidius celaquensis*						X	2500	1
*Geocharidius lencanus*						X	2500	1
*Geocharidius comayaguanus*						X	1300–2130	3
TOTAL SPP.	2	1	1	1	6	4		

Legend: CH, Chiapas Highlands; GC, Guatemalan Cordillera; HIH, Honduran Interior Highlands; MTZ, Motagua Fault Zone; SC, Sierra de los Cuchumatanes; SMC, Sierra Madre de Chiapas.

### Sympatric speciation

One common evolutionary trend among Anillina is syntopic miniaturization, a type of sympatric speciation that produces a number of related species differing in size and descendant from a common ancestor (Sokolov 2013). So, comparing average sizes of adults of *integripennis* group species in localities where sympatry has been recorded presented an interesting test of this idea. As noted above, we recognized six cases of sympatry (Fig. 22) involving the following species pairs (pairs marked by star are syntopic cases): *G. andersoni – G. zullinii* (Chiapas, Mexico), *G. antigua – G. minimus* (Volcano Agua, Guatemala), G. minimus – G. balini* (Volcano Atitlan, Guatemala), G. balini – G. jalapensis* (Mataquescuintla, Guatemala), G. lencanus – G. celaquensis* (Celaque National Park, Honduras), and G. comayaguanus – G. disjunctus* (Pico Pijol National Park, Honduras). These pairs of species can be grouped by the number of shared morphological characters into two categories: (1) a group of more closely related species that share two characters of male or female genitalia, namely the shape of the male ring sclerite and the shape of the female spermatheca; and (2) a group of more remotely related species that share only one character from either male or female genitalia. Data on size differences between species in all pairs are presented graphically as box-and-whiskers plots with regression lines (Fig. 23). For all pairs, we recorded the differences in averages of standardized body length between species. Our data support previous observations that the co-occurrence of taxonomically related anilline species in the same habitat is often accompanied by differentiation in their size (body length) (Pérez-Gonzáles and Zaballos 2013, Sokolov 2013). Perhaps the persistent (simultaneous) coexistence of two forms (a “larger” and a “smaller” form) in the litter reflects specific adaptations for living in only grossly overlapping microniches, which differ in some unknown parameters of substrate interspaces and thereby harbor different microbiotas. Hypothetically, slight divergence in niche preferences might result in divergence in target food preferences and decrease the number of contacts between representatives of “larger” and “smaller” forms. This, in turn, which could reduce competition between them and allow each to exploit resources more effectively.

At least in some cases, sympatry among anillines is a result of the dispersal of formerly allopatric taxa, typically in response to historical geological events and/or climate change. Interestingly, difference in sizes between a “larger” and a “smaller” species is evidently greater in pairs of more closely related species than in the pairs of more remotely related species. This difference can be seen visually in the slopes of regression lines and the means of the regression coefficients of these lines (Fig. 23, b). Unfortunately, the low number of observations does not allow us to analyze our data statistically and thereby evaluate how significant the observed differences between groups may be. We can only speculate about the origins and significance of differences between the two groups. For the present, we interpret our findings as reflecting differences in historical time at which each case of sympatry developed and, accordingly, by the length of time during which disruptive selection was occurring. We presume that, in the cases of the closely related species, we are dealing with intraspecific divergence, which was continuing for much longer times than in the cases of remotely related species, sympatry among which we consider a result of postspeciation dispersal, and thus of comparatively recent origin. In the latter case, interspecific divergence occurred over a much shorter time period, resulting in lesser differences in size between co-occuring species.

## Supplementary Material

XML Treatment for Geocharidius

XML Treatment for Geocharidius andersoni

XML Treatment for Geocharidius vignatagliantii

XML Treatment for Geocharidius zullinii

XML Treatment for Geocharidius antigua

XML Treatment for Geocharidius balini

XML Treatment for Geocharidius erwini

XML Treatment for Geocharidius gimlii

XML Treatment for Geocharidius integripennis

XML Treatment for Geocharidius jalapensis

XML Treatment for Geocharidius longinoi

XML Treatment for Geocharidius minimus

XML Treatment for Geocharidius celaquensis

XML Treatment for Geocharidius comayaguanus

XML Treatment for Geocharidius disjunctus

XML Treatment for Geocharidius lencanus
